# Construction of synthetic anti-fouling consortia: fouling control effects and polysaccharide degradation mechanisms

**DOI:** 10.1186/s12934-023-02235-7

**Published:** 2023-11-08

**Authors:** Ji Qi, Qicheng Zhou, Danlei Huang, Zhong Yu, Fangang Meng

**Affiliations:** 1 School of Environmental Science and Engineering, Sun Yat-sen University, Guangzhou, 510275 PR China; 2 Guangdong Provincial Key Laboratory of Environmental Pollution Control and Remediation Technology, Sun Yat-sen University, Guangzhou, 510275 PR China

**Keywords:** Membrane bioreactors, Membrane fouling, Biofilm formation, Synthetic anti-fouling consortia, Polysaccharide

## Abstract

The physical states and chemical components of bulk sludge determine the occurrence and development of membrane fouling in membrane bioreactors. Thus, regulation of sludge suspensions can provide new strategies for fouling control. In this study, we used “top-down” enrichment to construct a synthetic anti-fouling consortium (SAC) from bio-cake and evaluate its roles in preventing membrane fouling. The SAC was identified as *Massilia*-dominated and could almost wholly degrade the alginate solution (1,000 mg/L) within 72 h. Two-dimensional Fourier transformation infrared correlation spectroscopy (2D-FTIR-CoS) analysis demonstrated that the SAC induced the breakage of glycosidic bond in alginates. The co-cultivation of sludge with a low dosage of SAC (ranging from 0 to 1%) led to significant fouling mitigation, increased sludge floc size, and decreased unified membrane fouling index value (0.55 ± 0.06 and 0.11 ± 0.05). FTIR spectra and X-ray spectroscopy analyses demonstrated that the addition of SAC decreased the abundance of the *O*-acetylation of polysaccharides in extracellular polymeric substances. Secondary derivatives analysis of amide I spectra suggested a strong reduction in the *α*-helix/(*β*-sheet + random coil) ratio in the presence of SAC, which was expected to enhance cell aggregation. Additionally, the extracellular secretions of SAC could both inhibit biofilm formation and strongly disperse the existing biofilm strongly during the biofilm incubation tests. In summary, this study illustrates the feasibility and benefits of using SAC for fouling control and provides a new strategy for fouling control.

**Supplementary Information:**

The online version contains supplementary material available at 10.1186/s12934-023-02235-7.

## Introduction

Membrane-based processes, such as membrane bioreactors (MBRs), have emerged as one of a promising wastewater treatment technology owing to their high nutrient removal efficiencies and complete biomass retention [1]. However, membrane fouling remains a major challenge for the application of MBRs [2]. Typically, soluble microbial products (SMP) and extracellular polymeric substances (EPS) are the major contributors for membrane biofouling [3]. A considerable amount of biopolymers can be produced as a result of cell lysis and substrate metabolism, contributing to bio-cake formation on membranes. To develop strategies for fouling control, optimizing sludge characteristics, such as decreasing the amount of biopolymers in the sludge supernatant, is indispensable.

Polysaccharides and proteins can be consumed by the activated sludge or their producers. Numerous bacteria are involved in biomacromolecule metabolism, implying that enhancing the biodegradation of biopolymers in sludge can provide a new approach to fouling control [4]. Polysaccharides are important for the development of fouling because of their large size and gelling properties [5–8]. Polysaccharides can be degraded by commercial enzymes or by isolated strains. Previous reports have demonstrated that a commercial alginate lyase from *Sphingomonas* sp. can inhibit biofilm formation and disrupt existing biofilms (i.e., *Pseudomonas aeruginosa*) when being supplied exogenously [9]. Moreover, Okamura et al. [10] isolated a biofouling-degrading microorganism from activated sludge, *P. curvatum* HO1, which can degrade 30% of uronic acids in an SMP solution within 30 days of cultivation, reducing the filtration resistance of SMP solution. Note that the polysaccharides were not completely consumed. Nevertheless, the enzyme approach is challenged by the low stability and high operating cost [11, 12]. In addition, utilization of isolated strains is limited by the poor community coalescence in the mixed liquor and the high requirement of the cultivation process.

Compared to pure cultures, microbial consortia typically possess more diverse metabolic pathways and being adaptive to environmental stresses [13, 14]. Moreover, the microorganisms in a community can coordinate their specific activities through synergistic interactions [15]. Synthetic microbial consortia, with steerable functions, provide a promising way to improve operation stability and substrate availability. The “top-down” approach using elaborately selected environmental variables (e.g., substrate compositions, mean cell retention times, and redox conditions) shapes the microbiome by ecological selection, finally constructing a synthetic community with desired biological functions [16]. For instance, Geng et al. [17] enriched the alginate-degrading consortia dominated by *Bacteroides* (> 60%), which can effectively transform alginates. This methodological framework provides an ideal guidance for the establishment of polysaccharide-degrading consortia. The enhancement of biopolymer biodegradation may be more practical for membrane fouling in MBRs.

Inspired by previous efforts, we established and explored a “top-down” strategy for synthetic anti-fouling microbial consortium (SAC) enrichment and identified key microbial community members using Illumina high-throughput sequencing. The ability of the SAC to biodegrade polysaccharides was characterized, and its efficiency in preventing membrane fouling was assessed via biofilm tests. Overall, this study advances our understanding of biofouling control in MBR operations by providing a protocol for polysaccharide-degrading consortia.

## Materials and methods

### Enrichment of SAC

The enrichments were inoculated with bio-cake, which were collected from the fouled membranes of a local MBR plant in Zhuhai, China. The consortia were incubated with sodium alginate (SA) as the sole carbon source in a defined mineral salt medium (MSM, Table S1, Table S2). To determine the minimum carbon source concentration for enrichment and improve microbial consortium selectivity, the contents of SMP and EPS in bio-cakes were used as the basis. The carbon source concentrations were then gradually increased to further enhance the metabolic activity of the microbial community, leading to increased production of enzymes and metabolites. The initial concentration of SA was set at 100 mg/L, and the concentration was gradually increased after every 3 passages (500, 1,000, and 2,000 mg/L). The consortia were grown in a bottle flask with a working volume of 50 mL during the enrichment cultivation. The passaging was performed every 7 days by centrifuging the incubated bacteria at 3,500 rpm for 5 min and washing three times with PBS solution (pH = 7.2). All enrichment consortia were incubated at 30^o^C for 4 weeks in a constant-temperature incubator (150 rpm).

### Characterization of microbial consortia

The DNA of SAC was extracted using the DNeasy PowerSoil Kit (Qiagen, Inc., The Netherlands), following the manufacturer’s instructions. The V3-V4 regions of microbial community 16 SrRNA were amplified with the primers 338 F and 806R. Detailed information on the PCR amplification is shown in Text S1. Then, paired-end sequencing with equimolar purified amplicons was performed on an Illumina HiSeq250 platform. Raw sequences were processed by QIIME2.0 software [18]. The low-quality sequences were removed from the raw sequencing data, and the high-quality sequences were then analyzed with the Divisive Amplicon Denoising Algorithm (DADA2) pipeline to generate amplicon sequence variants (ASVs) [19]. Classification assignment for each representative ASV was annotated based on the Silva database with a confidence threshold of 80%.

### Biodegradation assay of microbial consortia

The assay was conducted to investigate the polysaccharide degradation capability of SAC. Initially, the consortia were inoculated into 100 mL of MSM and incubated aerobically in the rotator shaker (150 rpm, 30^o^C) for the mid-exponential phased phase (OD_595_ = ~ 0.17). Then, the biomass was harvested by centrifugation (3,500 rpm, 10 min), washed with PBS solution (pH = 7.2) thrice, and resuspended in 200 mL fresh MSM with 1,000 mg/L SA as the only carbon source. All the experiments were performed in a 250 mL bottle flask sealed with parafilm and incubated in a rotator shaker (150 rpm) at 30^o^C, sampling at two-hour intervals. The supernatant sample was filtered with 0.45 *µ*m membranes. The biomass concentrations were measured according to the standard method [20]. After 74 h of aerobic incubation, the polysaccharide concentrations were measured to determine the biodegradability of SA. Then, the degradation rate was normalized against biomass concentrations, and the first-order kinetics was employed to fit the biodegradation rate.

### Short-term filtration tests

**Fig. 1:**
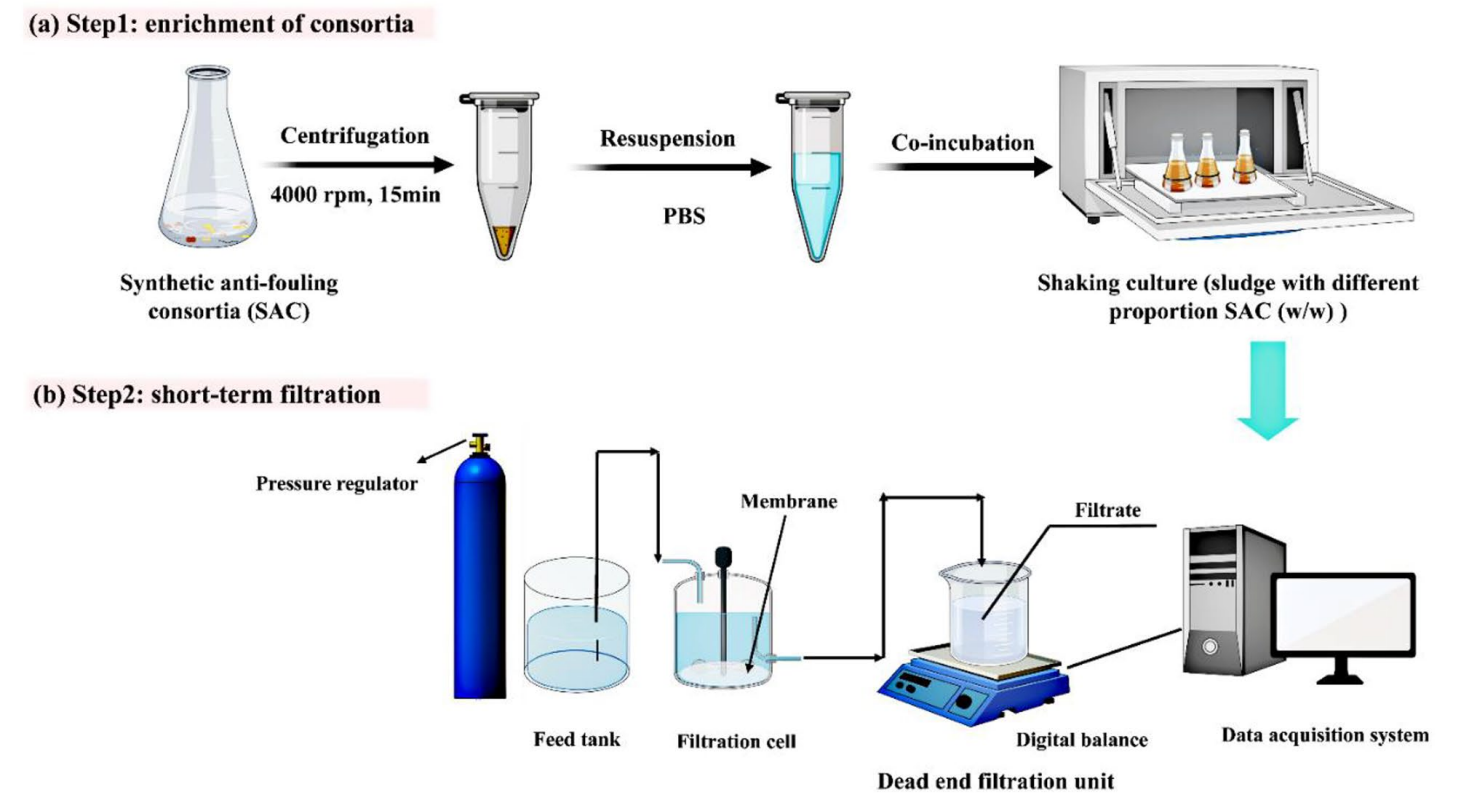
Overview of the experimental process and approach. **(a)** Step1: Co-incubation of sludge with different proportions of bacteria (w/w%). **(b)** Step 2: Short-term filtration was conducted to assess the effects of SAC on the membrane fouling

The activated sludge was collected from a lab-scale membrane bioreactor with a constant volatile suspended solids concentration (VSS) of 3,000–3,500 mg/L. The sludge samples were washed with 0.05% NaCl solution twice to remove the residual nutrients. The SAC consortia were added to the washed sludge with varying dosages (0%, 0.1%, 0.5%, 1%, 2%, 5%, 10%, w/w) and incubated for 72 h (Fig. 1, Step 1). Each experiment was implemented in triplicate.

A dead-end stirred cell (MSC100, Mosul Co., Shanghai, China) with an effective filtration area of 12.5 cm^2^ was applied to assess the membrane fouling potentials of the SAC-dosed sludge (Fig. 1, Step 2). Prior to the filtration, the pristine membrane (PVDF, 0.1 *µ*m) was immersed in deionized water for 24 h and filtered deionized water until the permeate flux remained stable. In the filtration process, 50 mL of the SAC-dosed sludge sample was loaded into the cell, and the filtration was fixed at a constant pressure of 50 kPa and a constant stirring speed of 500 rpm. The permeate volume was recorded with a digital balance connected to a computer. The unified membrane fouling index (UMFI) was used to evaluate the membrane fouling potential of different sludge mixtures [21]. A higher UMFI value indicates greater fouling potential during the membrane filtration process. The calculation method of UMFI is provided in Text S2.

### Biofilm disruption and inhibition assay

To evaluate the active species responsible for SA degradation, cell fractionation was performed (Text S3). For biofilm disruption assays, *P. aeruginosa* (PAO1) was grown in liters of Lauria-Bertani (LB) broth at 30^o^C over 24 h with shaking at 150 rpm. The bacterial cultures were standardized to OD_595_ of ~ 0.1 with Luria-Bertani broth (LB) and added to sterile 96‐well microtiter plates with four replicates. Then, the cultures were incubated statically at 30^o^C for 24 h to allow for biofilm formation. After incubation, nonadherent cells were disassembled by deionized water. The wells were added with 100 *µ*l of varying concentrations extracellular secretions of SAC (16.5 ± 0.75–165 ± 7.52 mg/L). After 24 h of incubation, crystal violet (CV) assay with minor modifications was used to quantify the biofilm biomass [22, 23]. The bacteria in wells were stained with 150 *µ*l of 0.5% CV for 20 min, rinsed with deionized water. The remaining satin was solubilized by additional 33% acetic acid for 15 min, after which the absorbance was measured at 595 nm using the microplate reader (Multiskan FC, Thermo Scientific).

For biofilm inhibition assay, PAO1 was grown over 24 h and then adjusted to OD_595_ of ~ 0.1 to induce the biofilm formation. Diluted culture (90 *µ*l) was added to sterile 96-well microtiter plates, and extracellular secretions of SAC at different concentrations were added in 10-*µ*l aliquots to the final volume of 100 *µ*l. The cultures were incubated statically for 24 h at 30^o^C to allow for biofilm formation. Then, CV assay was used to evaluate the inhibition effects of biofilm. All experiments were completed in triplicate. Besides, to determine whether the disruption of biofilm due to an indirect effect of inhibition of bacterial growth, we set up a bacterial growth assay (Text S4).

### Physico-chemical analysis

After 72 h incubation, the EPS of SAC-dosed sludge was extracted using the heating method [24]. In brief, the culture sample was centrifugated for 15 min at 3,500 rpm, 4^o^C. Then, the pellet was resuspended to the original volume with 0.05% NaCl solution and heated in a water bath for 30 min at 60^o^C. After that, the sample was centrifugated again for 15 min at 12,000 rpm, 4^o^C, and the supernatant after filtering with 0.45 *µ*m membranes was EPS. The concentrations of polysaccharides and proteins were measured using phenol sulfuric acid and Lowry methods, respectively.

The EPS solution or alginate solution was freeze-dried at -80^o^C for 3 days (Alpha 1 − 4 LSCplus, Christ Co., Germany). Afterward, the dried powder was then analyzed using a Fourier Transform infrared spectroscopy (FTIR, Thermo Fisher Scientific Co., USA). The FTIR spectrum consisted of 16 scans over the range of 4000 − 700 cm^− 1^ with a resolution of 2 cm^− 1^. To investigate the protein secondary structure and potential changes upon SAC dosage, the amide I (1600–1700 cm^− 1^) region of the FTIR spectrum was further analyzed by assuming a Gaussian line shape for each peak using PEAKFIT v4.12 software. Additionally, the 2D-CoS analysis was conducted to analyze the structural change of alginate molecules during the biodegradation assay. A series of time-dependent FTIR spectra were therefore collected. Based on the work of Noda and Ozaki, 2D-FTIR-COS was involved to generate synchronous and asynchronous spectra, details about the 2D-FTIR-COS calculation were provided in Text S6.

The elemental composition of the EPS samples was analyzed by X-ray photoelectron spectroscopy (XPS, ESCALAB 250, Thermo Fisher Scientific Co., UK). XPS measurement was conducted using Al *Kα* radiation (1486.6 eV). Concentrations of dissolved organic carbon were analyzed using a total organic carbon analyzer (TOC, TOC-L, Shimadzu, Japan). The above measurements were repeated 5 times. The particle size distribution was determined by DLS analysis (Malvern 3000, UK). Scanning electron microscopy (SEM, JEOL JEM-2010 h, Japan) was conducted to compare the biofilm structure after the dead-end filtration. The zeta potential of each sample was analyzed using a zeta analyzer (Nano ZS90 Malvern Corp., UK). The membrane water contact angle (WCA) was measured using a contact angle analyzer (DSA100E, Germany), the details of the measurements were available in the Text S7.

## Results and discussion

### Community composition of the SAC

**Fig. 2:**
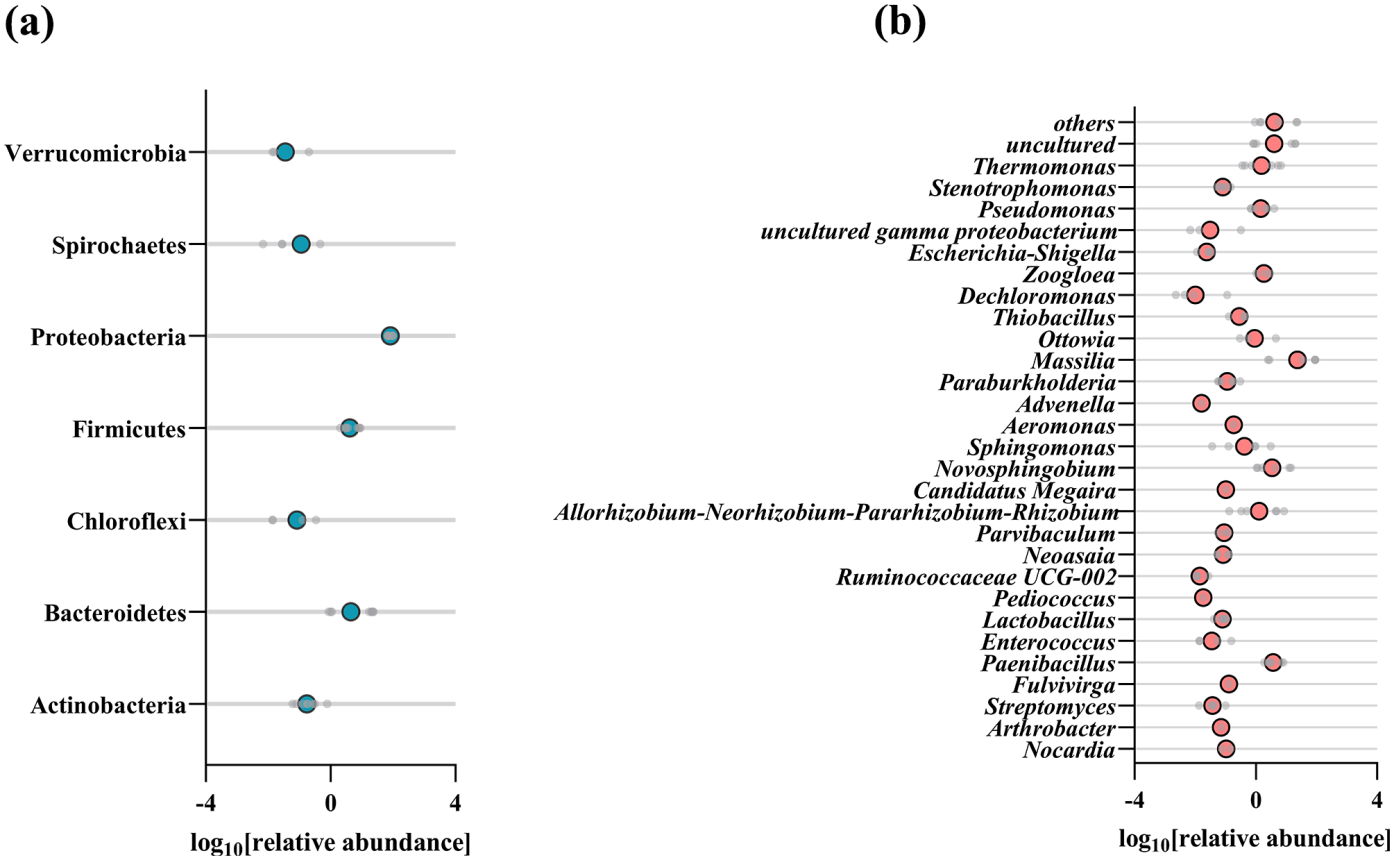
16 S rRNA sequencing data of the SAC. Relative abundances of the abundant bacterial clades at the level of phylum **(a)** and genus **(b)** in the SAC. Relative sequence abundances are obtained based on 16 S rRNA V3, V4 gene amplicons

The changes in microbial community composition over incubation indicate the selective enrichment of specific taxa (Fig. 2). At phylum level, Proteobacteria and Bacteroidetes were the two dominant bacterial populations in the consortia (averaged at 90%), and they remained stable during the enrichment process. At genus level, *Massilia* (averaged at 35%) was the most abundant genus in SAC, followed by *uncultured Chitinophagaceae* (averaged at 23%), *Paenibacillus* (averaged at 6.95%), and *uncultured Burkholderiaceae* (averaged at 7%).

The genus *Massilia*, which is ubiquitous in agricultural soils supplemented with chitin, degrades macromolecular chitin under aerobic conditions [25]. In addition, Han et al. [26] isolated a microorganism, UMI-21 (phylogenetically related to the *Massilia* sp.), that could utilize starch as the sole carbon source. A strain belonging to the genus *Paenibacillus* isolated from the root soil of cypress was reported to effectively degrade and utilize marine-derived polysaccharides, such as agar, alginate, and chitin [27, 28]. Overall, these previous findings suggest that the SAC enriched in the current study was composed of various candidates with a strong capability to degrade polysaccharides.

### Biodegradation of alginates by the SAC

**Fig. 3:**
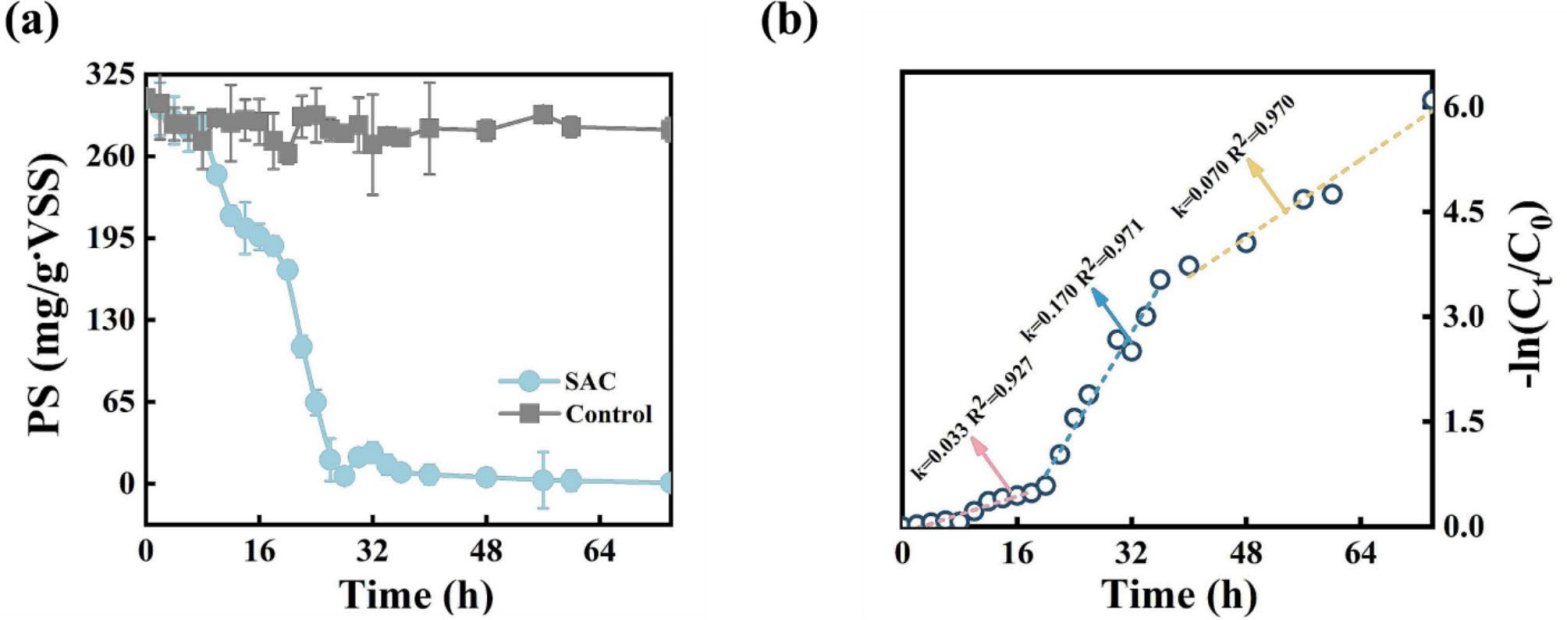
Sodium alginate biodegradation by SAC under aerobic conditions. **(a)** Removal of SA by SAC over 74 h. **(b)** first-order kinetic model foe degradation of SA. All experiments were conducted in triplicates with an initial OD_595_ of 0.17 for the SAC.

The SAC degradation capability of SAC was evaluated using an SA solution (1,000 mg/L). Under aerobic conditions, the enriched microbial consortia were well capable of utilizing SA as the sole carbon source, i.e., the polysaccharide concentration decreased from 305 ± 6.05 to 0.68 ± 2.74 mg/g⋅VSS (Fig. 3). The overall SA degradation process within 74 h was divided into three phases, as described by the first-order kinetics (R^2^ > 0.93) shown in Fig. 3b. The kinetic constants for biodegradation were determined to be 0.03, 0.17, and 0.07. These differences might correspond to the initial adaption of SAC to the SA solution, the rapid consumption of alginate, and the deficiency of nutrients. Ji et al. [29] reported a pure culture that took 5 d to degrade SA, resulting in more than 20% of residual alginates. Zhang et al. [30] reported that the alginates could be completely consumed for methane production by anaerobic alginate hydrolytic bacteria within 10 d. Overall, the enriched SAC from the fouled membranes in the study exhibited a high SA conversion rate in a short time.

**Fig. 4:**
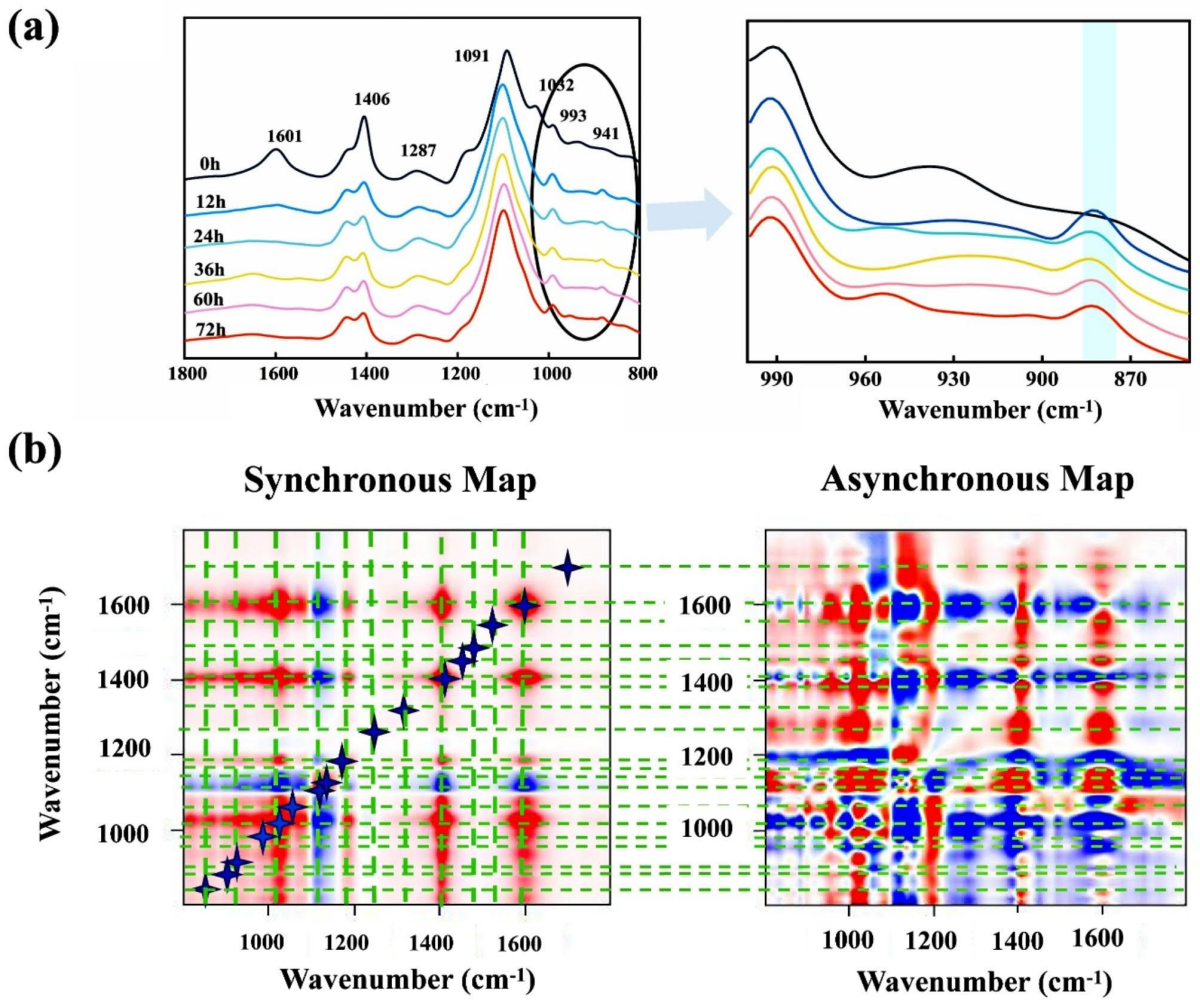
FTIR spectra and 2D correlation spectroscopic (2D-COS) analysis. **(a)** FTIR spectra (1800 − 800 cm^− 1^) of SA degradation within 74 h. **(b)** Synchronous and asynchronous 2D correlation maps generated from FTIR spectra of SA biodegradation within 74 h. The red indicates a positive correlation, and the blue represents a negative correlation. Darker color indicates a higher intensity, thus a stronger positive or negative correlation. The signs of corresponding autopeaks (at the diagonal) and cross peaks (off the diagonal) in both synchronous and asynchronous maps are presented in Table S3

To investigate the functional response of the SA molecules to the SAC biodegradation, the FTIR spectra were obtained at varying degradation times (Fig. 4). The FTIR peaks located at 1650–1600 and 1500–1400 cm^− 1^ were associated with the presence of the vibrations of C = O and -COOH, respectively [31–33]. The weak band located at approximately 1300 cm^− 1^ was attributed to the C − C−H or O − C−H [34]. The sharp peaks located at 1250–1150 and 1100–1000 cm^− 1^ were assigned to the vibrations and stretching of C − O−C bonds in alicyclic ether, respectively [35, 36]. In addition, the two peaks at 1000–900 and 900–800 cm^− 1^ were attributed to the C–O stretching of uronic acids as well as C − H deformation [37]. An obvious change in the FTIR spectra was the generation of new peaks located at approximately 1650 cm^− 1^ with the increasing incubation time, which was related to the COOH stretching of the free carboxylic acid groups in SA [38]. The formation of carboxyl groups suggests that the glycosidic bonds are broken during the degradation process, changing the structure of the end residue and forming the C = O groups [33, 39]. In addition, a profound change was observed in the 1450 − 1000 cm^− 1^ region, with a stronger vibrational spectral intensity for symmetric vibrations of COOH, and a weak stretching of C–O–C located at approximately 1447 and 1103 cm^− 1^, respectively. In addition, the open structure of the dissociated cross-linked SA provided large conformational freedom for the functional groups, increasing their spectral intensities, such as those of uronic acids and C − H bonds located at 993 and 885 cm^− 1^ [40]. The microbial pathways responsible for SA metabolism are initiated by polysaccharide lyases, which cleave the polysaccharide backbone to produce uronic acids with 4,5-unsaturated nonreducing ends [41]. Therefore, the exposed structures of the dissociated SA were further oxidized and the spectral intensities of the functional peaks decreased (e.g., the peaks at 993 and 885 cm^− 1^).

2D-FTIR-CoS analysis was conducted to provide credible information concerning on the response of functional groups of the alginates to SAC degradation (Fig. 4b). Overall, 17 characteristic peaks (1725, 1650, 1604, 1406, 1317, 1294, 1205, 1161, 1137, 1122, 1103, 1056, 1020, 991, 956, 916, and 885 cm^− 1^) were observed along the diagonal line. The peaks at 1604, 1406, 1056, and 1020 cm^− 1^ had the highest intensities, followed by the peaks at 1161, 1137, 1122, 1103, 991, 956 and 885 cm^− 1^. However, 1725, 1650, 1294, 1205, 1317, and 885 cm^− 1^ had the lowest intensities. This implied that the COOH and C − O−C groups were more sensitive to biodegradation than the other characteristic groups. Most of the cross peaks in the synchronous map had positive signs (Table S4), indicating that these characteristic groups underwent synchronously changes during the biodegradation process.

According to the Noda’s rule described elsewhere [42], the biodegradation of SA molecules within 74 h followed the sequence: the asymmetric vibration of COOH groups, symmetric vibration of COOH, C − O−C stretching of uronic acids and C − H deformation (1604, 1317, 991, 885 cm^− 1^) > asymmetric C − O stretching of COOH, symmetric vibration of COOH and C − O stretching of uronic acids (1650, 1406, 916 cm^− 1^) > vibration of C − O−C bonds (1294, 1137, 1122 cm^− 1^) > antisymmetric C − O−C stretching (1056 cm^− 1^) > vibration of C − O−C bonds and C-O stretching of uronic acids (1103, 1018, 956 cm^− 1^) > vibration of C − O−C bonds (1205 cm^− 1^) > asymmetric C − O stretching of COOH and vibration of C − O−C bonds (1317, 1161 cm^− 1^). This sequential order demonstrates the biodegradation mechanisms of SA. The damages or changes in COOH, C − O−C, and hydrogen bonds (1604, 1317, 885 cm^− 1^) weaken the SA molecular network [43, 44]. These oligosaccharide products can be converted into monomers [45], resulting in the yield of C − O−C rupture (991 cm^− 1^) and O − C−H deformation (1,406 cm^− 1^). These products are then degraded in a stepwise manner to the intermediates 4,5-unsaturated monouronates, ultimately producing the key metabolites of 2-keto-3-deoxygluconate glyceraldehyde triphosphate (G-3-P) and pyruvate (Figure S1) [41, 46].

### Roles of SAC in mitigating membrane fouling

**Fig. 5:**
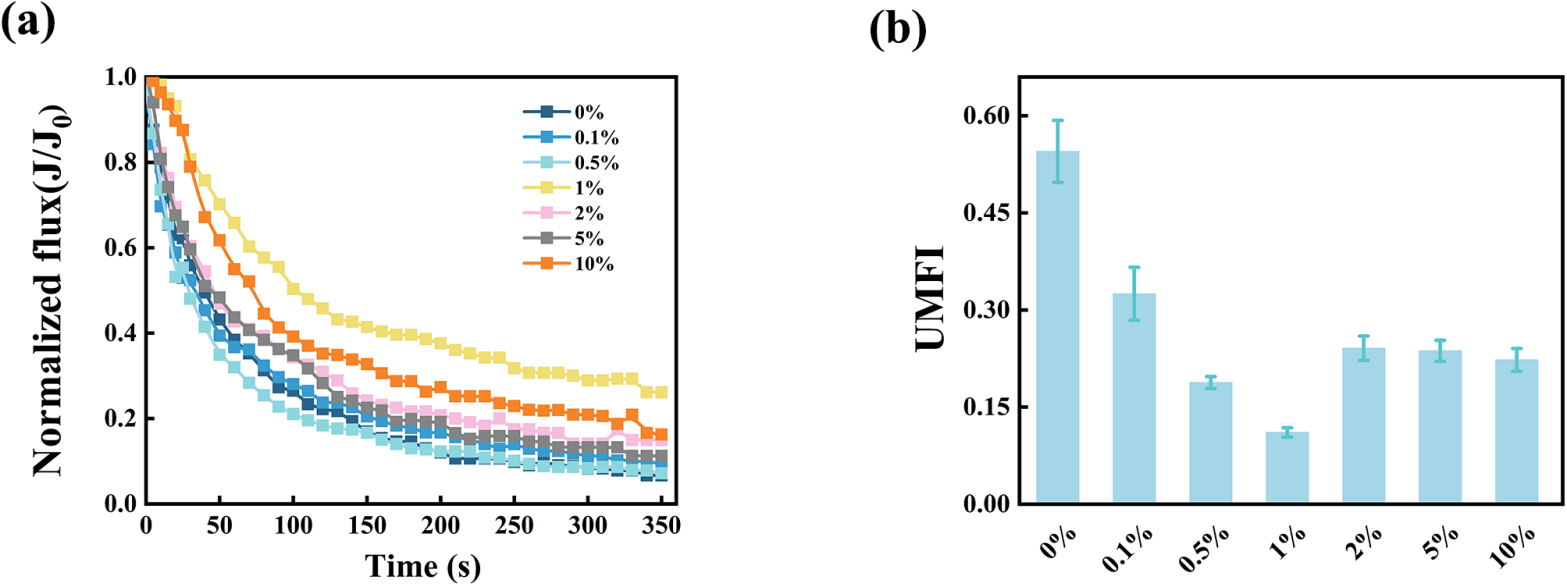
Fouling control effects of the SAC-incubated mixed liquor. **(a)** Filtration curves (normalized flux vs. time) of incubated sludge with different dosages of SAC. **(b)** Variations of the corresponding UMFI in dead-end filtration

We investigated the efficacy of the microbial consortia in controlling fouling by conducting a series of filtration tests. The decline in permeate flux was monitored (Fig. 5a). The permeate flux of the control group decreased rapidly, reaching a final *J*/*J*_0_ of approximately 15.1%. The sludge with the SAC dosage exhibited a slower decline in the permeate flux during the filtration process; for example, the final *J*/*J*_0_ of the 1% inoculation group was 26.7%. The improved filtration ability of the SAC-incubated sludge was further verified using the UMFI (Fig. 5b). For instance, the control and 1% inoculation groups had a UMFI of 0.55 ± 0.06 and 0.11 ± 0.05, respectively. However, an excessively high SAC (> 2%) led to severe membrane fouling. As characterized by the SEM, in the control group (0% inoculation), a large amount of sludge was found to adhere to the membrane surface, while a few microorganisms were observed on the membrane surface in 1% inoculation group (Figure S2).

It was found that the SAC-incubation decreased the TOC concentrations significantly in sludge supernatant significantly (ca. 46%, Figure S3a). Meanwhile, the polysaccharide content in the SMP showed a downward trend, likely owing to the increased catabolic capacity of the enriched microbial consortia (Figure S3b). Membrane fouling is a long-term, dynamic process during MBR operation. The SMP plays a significant role in initial fouling before the TMP jump [45]. Therefore, the improved polysaccharide biodegradation by the enriched SAC in this study could effectively alleviate the formation of a gel layer on the membrane surface during the MBR operation.

Large sludge flocs accounted for a higher proportion in the inoculation group than in the control group (Figure S4a). The volume weighted mean diameter *D* [3, 4] significantly increased slightly as SAC was dosed, i.e., from 54.3 *µ*m to 68.2 *µ*m. Smaller particle-sized flocs have been proven to be predominant foulants because of the preferential deposition on the membrane surface [48]. Combined with the above results, the presence of floc-forming bacteria (i.e., *Zoogloea*) in the SAC supported larger flocs in it. After introducing the SAC into the sludge system, its members can secrete specific biomolecules. The newly produced EPS may have different chemical compositions and molecular weight compared to the existing sludge EPS, impacting the overall structure of the sludge. In this study, the EPS compositions (Figure S4b) as determined PN/PS ratio of the various groups were also different from each other (0.78 ± 0.03, 1.25 ± 0.03, 1.70 ± 0.04, 1.95 ± 0.06, 1.81 ± 0.05, 1.24 ± 0.07, 1.71 ± 0.17, respectively). The increase in the ratio of PN to PS can induce an increase in the hydrophobicity of sludge and the biological flocculation ability [47]. Thus, the newly produced EPS may have a different chemical composition, and molecular weight compared to the existing sludge EPS, impacting the overall structure of the sludge. However, a previous study has revealed that the EPS produced by the deposited microbial cells was a major foulant after the TMP jump [10]. Therefore, an increase in EPS secreted by a higher content of SAC (10%) could also lead to a reduction in the filterability of sludge, flux decline and increases in cake resistance (Fig. 5a, Figure S4b).

Moreover, the sludge flocs presented a higher WCA against water and a lower zeta potential value in the inoculation group (Table S5), suggesting a more hydrophobic cell surface and a weaker repelling force between sludge. The weaker the mutual attraction between sludge particles with lower absolute negative charge and the negatively charged membrane surface, the less likely adhesion and blockage will occur between the sludge and membrane. This can potentially reduce the risk of membrane fouling caused by negatively charged sludge. Besides, the high WCA and low zeta potential value of the sludge flocs in the inoculation group imply that the cell surfaces exhibit increased hydrophobicity, leading to weak repulsive forces between the sludge particles. As a result, the interactions among these particles tend to aggregate and form flocs.

### Response of EPS compositions to SAC incubation

**Fig. 6:**
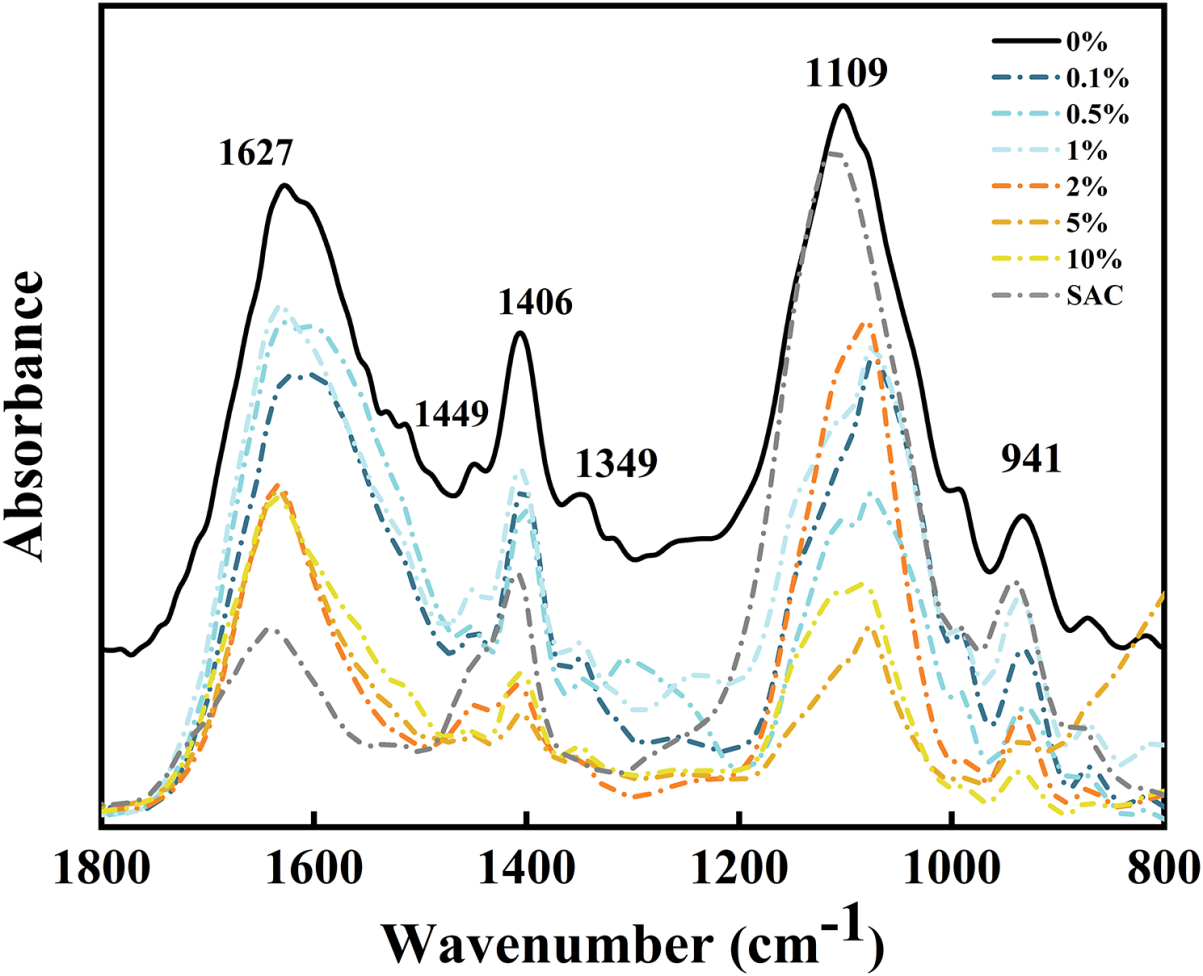
FTIR spectra (1800 − 800 cm^− 1^) of EPS extracted from sludge and the SAC-incubated sludge. The specific differences in FTIR spectra are shown in Table S6

EPS production plays a crucial role in biofilm formation [48–50]. The FTIR spectra of EPS samples extracted from the sludge after 72 h of incubation in the presence and absence of SAC were mainly concentrated at 1800 cm^− 1^–800 cm^− 1^. Several characteristics were observed in the FTIR spectra, demonstrating the presence of proteins and polysaccharides in the EPS (Fig. 6, Table S6). The amide I (~ 1630 cm^− 1^), amide II (~ 1550 cm^− 1^), and amide III (1300–1200 cm^− 1^) were detected in FTIR [51]. In addition, the peaks at 1020 cm^− 1^ and 920 cm^− 1^ are associated with the symmetric and asymmetric stretching of C–O–C bonds in the pyranosyl ring and the C–O–C stretching of uronic acids, respectively [52, 53].

Differences were observed in the functional groups of the EPS for all samples. An obvious change in the FTIR was the appearance of bands located at 1550 cm^− 1^ and 1300 cm^− 1^ over SAC dosage and incubation. The amide II peak at 1550 cm^− 1^ was absent in the control group. Moreover, the absorption of the amide III band exhibited a stronger spatial dependence. This phenomenon may be related to the sensitivity of the band to different growth environments. Therefore, the band was affected by the local conformational change accompanying the transition of proteins [54]. In addition, the absorbance intensity of the peaks at ~ 1100 and ~ 920 cm^− 1^ was stronger in the control group, indicating relatively enriched *O*-acetylated carbohydrates compared to those in the inoculation group [55]. Uronic acids (920 cm^− 1^) are an indicator of alginate-like polysaccharides, which possess a strong gelling property [56–58] Stubborn uronic acids are mainly responsible for irreversible fouling potential, because of their contribution to pore blocking [59]. This suggests that the SAC effectively degraded polysaccharides by damaging their functional groups.

Sludge characteristics are closely related to the secondary structures of surface proteins, which play a key role in membrane fouling [60]. A detailed analysis of EPS secondary structures was conducted by focusing on the derivative spectra (Figure S5 and Table 1). Amide I in the EPS contained aggregated strands (1610–1625 cm^− 1^), *β*-sheets (1630–1640 cm^− 1^), *α*-helices (1648–1657 cm^− 1^), 3-turn helices (1659–1666 cm^− 1^), and *β*-antiparallels (1680–1695 cm^− 1^). The random coil of the secondary structure was absent from the EPS. *β*-sheets were more dominant than other secondary structures in the amide I band of all EPS samples. A clear difference in the absorption of the amide I band was observed between the incubated and control groups. *β*-sheets (34.0–42.1%) were the most dominant fraction in the amide I band. The proportion of antiparallel *β*-structures, which is an indicator of membrane fouling, was negatively correlated with the UMFI values (Figure S6, *P* < 0.05). Additionally, the degree of tightness (*α*-helices/(*β*-sheets + random coils)) is generally considered to indicate the compositional characteristics of the sludge protein structure [61]. A reduced degree of tightness indicates a loosened protein structure that promotes sludge aggregation [62]. Here, the *α*-helix/(*β*-sheet + random coil) ratio decreased as the sludge was dosed with SAC (from 0.56 to 0.42), which was consistent with the particle size of sludge flocs.

**Table 1:** Band assignments for the protein secondary structure of EPS from SAC-incubated sludge from derivative spectra and curve fitting

Group	Aggregated strands (1610–1625)	*β*-sheet (1630–1640)	*α*-helix (1648–1657)	3-Turn (1659–1666)	*β*-anti (1680–1695)	Tightness degree
0%	10.3%	34.0%	19.1%	28.5%	8.18%	0.57
0.1%	10.4%	39.5%	18.7%	25.8%	5.62%	0.47
0.5%	6.08%	38.1%	21.0%	28.7%	6.13%	0.55
1%	12.9%	42.1%	17.6%	22.7%	4.67%	0.42
2%	10.7%	38.7%	19.3%	25.6%	5.70%	0.50
5%	9.84%	38.3%	18.7%	26.8%	6.36%	0.48
10%	10.3%	37.1%	20.7%	25.0%	7.31%	0.56

**Fig. 7:**
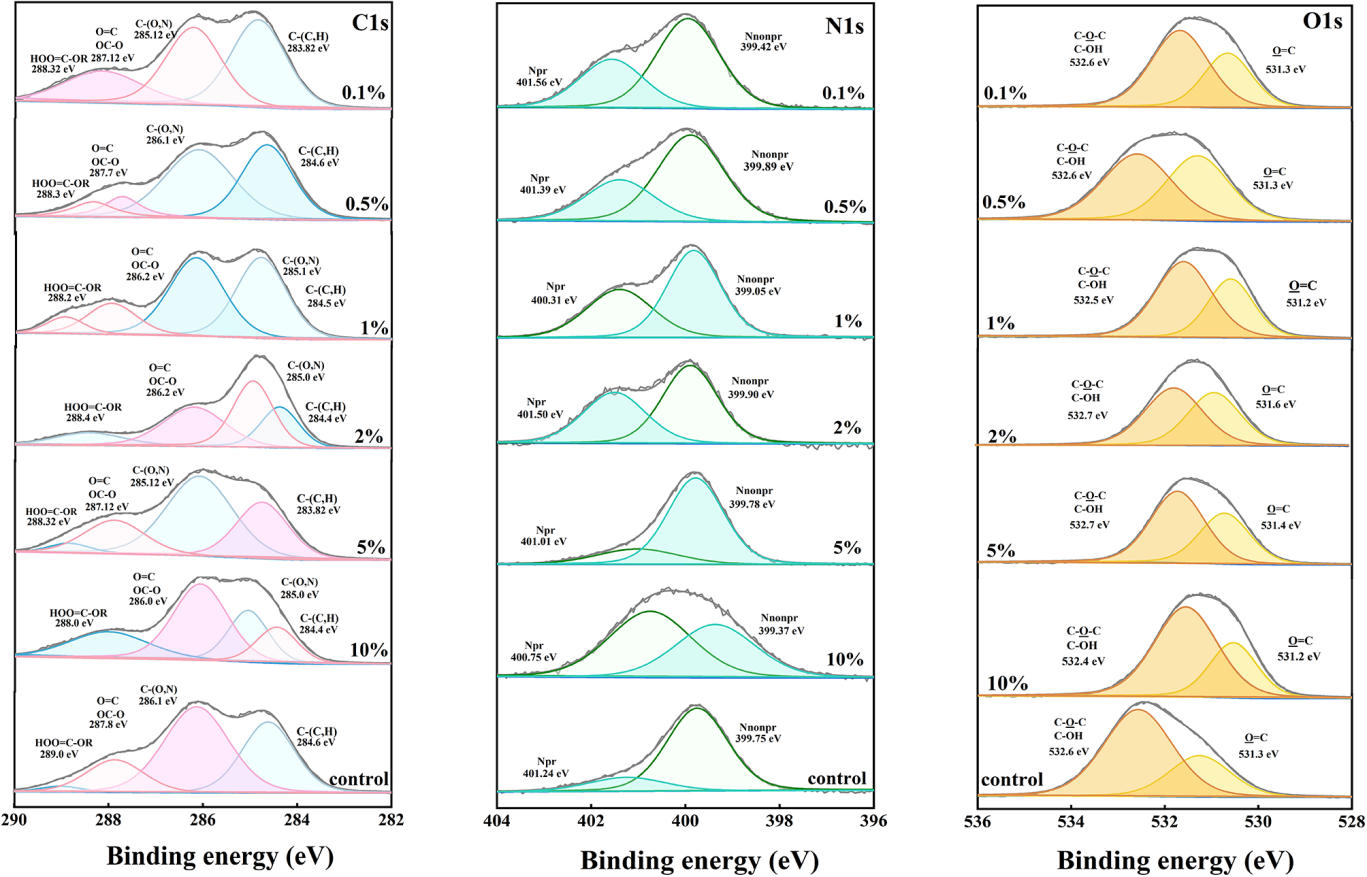
XPS spectra of EPS and high resolution scans of C, N, and O spectra, respectively

Figure 7 presents the XPS spectra of the EPS collected in the presence and absence of SAC. High-resolution scans of C1s, N1s, and O1s served as the first step in obtaining detailed information on chemical functionality [63]. The C 1s peaks at approximately 284.4, 284.9, 285.3, and 286.1 eV are attributed to C–(C,H), C–(O,N), O = CO, and O = C, respectively [64]. The O 1s peak at approximately 531.5 eV can be assigned to O = C groups, such as carboxylate, carbonyl, ester or amide groups. The peak at 532.5 eV corresponds to O–(C,H) as alcohol, hemiacetal, or acetal groups [65]. These results indicated the presence of polysaccharides, carboxylates, and carboxyl groups. In addition, the N 1s peak resolved into two-component peaks. The peak at 399.9 eV is mainly attributed to the Nnonpr from the amines and amides; and the peak of 400.1 eV originates from the Npr [66], which is commonly found in amino sugars and amino acids such as lysine and arginine.

Acidic carbohydrates contributed more to EPS when SAC was added (Table 2), as evidenced by the higher O_531.3_ molar ratios. The presence of SAC (1%) decreased the polysaccharide concentrations (O_532.7_ molar ratios) by ~ 30% in the EPS. The decrease in the elemental (O/C) ratios of the sludge with increasing SAC dosage was quantitatively in line with the trends of the FTIR data. These XPS results are supported by the FTIR bands analysis between 1200 and 900 cm^− 1^ in Fig. 6.

**Table 2:** N and O atomic ratios and functional groups with respect to C from the high resolution XPS spectrum used to quantify the EPS extracted from SAC-incubated sludge

Group	Elemental composition (molar ratios with respect to total carbon)		Chemical function (relative ratio (%))							
	O/C	N/C	C-(C,H)	C-(O,N)	O = CO- -C-O	O = CHO O = C-O-R	Nnonpr	Npr	O = C C-P = O	HO-C C-O-C
0%	0.54	0.11	19.1	28.6	9.62	1.25	5.07	1.44	9.49	22.1
0.1%	0.54	0.17	21.9	25.5	10.2	0.72	5.84	3.25	18.3	10.2
0.5%	0.51	0.20	15.2	27.2	10.2	1.89	7.29	3.64	13.1	14.6
1%	0.53	0.20	19.2	21.3	7.16	2.93	5.83	4.03	10.5	15.7
2%	0.49	0.14	24.1	10.3	3.76	1.68	3.78	1.80	12.7	15.1
5%	0.48	0.16	28.9	17.5	11.0	0.68	6.94	1.82	11.0	16.7
10%	0.50	0.19	19.2	20.3	7.16	2.93	3.45	4.42	9.21	20.0

### Effects of SAC on biofilm formation

**Fig. 8:**
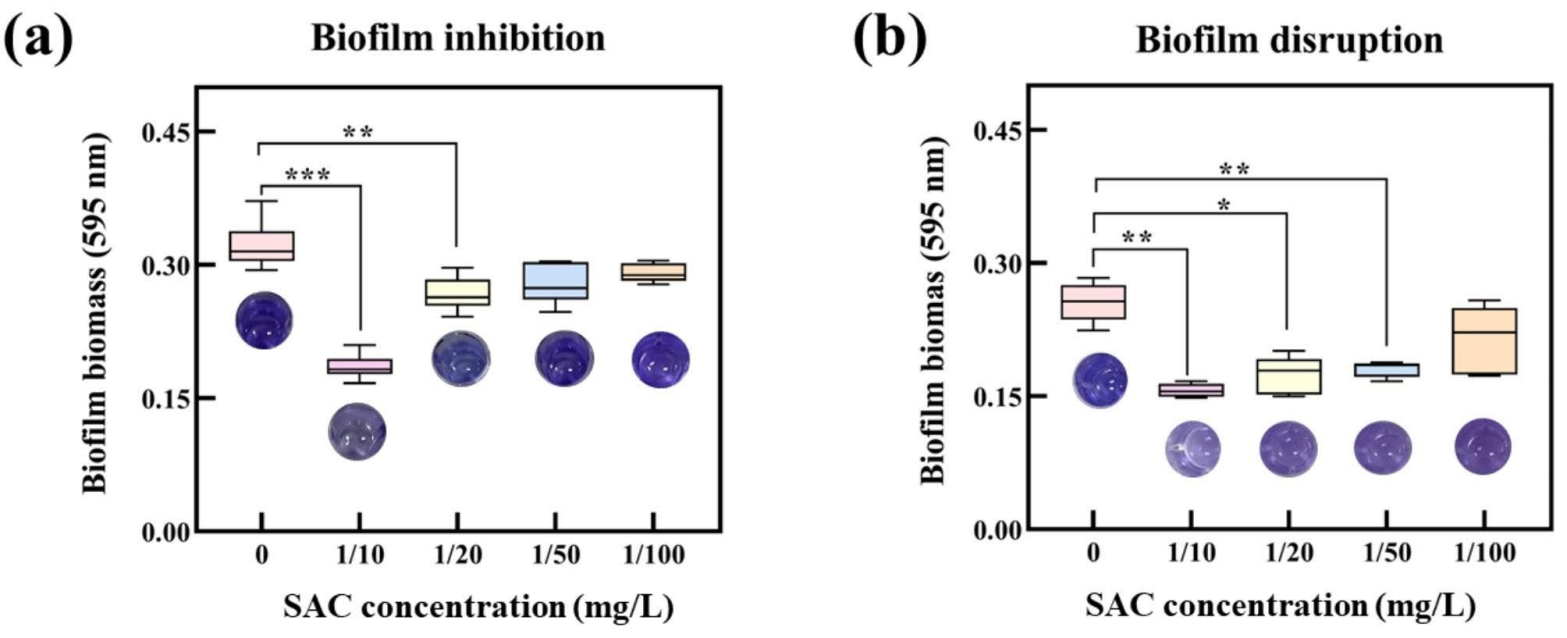
**(a)** The extracellular secretions of SAC prevent the biofilm formation of *P. aeruginosa* (PAO1). **(b)** The extracellular secretions of SAC disrupt the existing biofilm of PAO1. Crystal violet (CV) staining was used for biofilm quantification. Each data implies the mean from independent assays of *n* = 5 microtiter plate wells. Error bars denote SEM. ** *P* < 0.01, *** *P* < 0.001

To determine which fractions of the consortia were responsible for polysaccharide degradation, active compounds of the SAC were isolated. As shown in Figure S7, approximately 46% of SA-degrading efficiency was found in the extracellular secretions, while both the periplasmic and intracellular fractions of SAC had little impact on SA removal (~ 21%) after 48 h of cultivation, providing evidence that SA degradation was an extracellular process. We hypothesized that exogenous addition of the extracellular fraction of SAC to the biofilm would lead to the hydrolysis of exopolysaccharides, thus disrupting the established biofilm. To evaluate the biofilm disruption, we used PAO1 as a model strain for the investigation of biofouling. A CV assay was used to quantify the effect of extracellular secretions on the biofilm biomass. The 24-h treatment of the PAO-dependent biofilm with SAC resulted in a ~ 40% reduction in biofilm biomass. The activity of the extracellular secretions was dose-dependent (*P* < 0.001). The extracellular secretion of SAC showed a continuous decrease in biofilm biomass with increasing SAC concentration. A 24-h treatment of the established biofilm with extracellular secretions at a concentration of 16.5 ± 0.75 mg/L resulted in disruption of 50% of the biomass (Fig. 8a).

Because the extracellular secretions of SAC were effective in disrupting the established biofilms, we sought to determine whether the employment of this secretion in bacterial culture could serve as a prophylactic way to inhibit biofilm formation. To evaluate the biofilm-inhibiting performance, the extracellular secretion of SAC was added to the culture media containing PAO1 during inoculation (Fig. 8b). The addition of extracellular secretions of SAC under biofilm-forming conditions resulted in ~ 41% inhibition of biofilm biomass over 24 h at 30 ℃. In addition, the bacterial growth of PAO1 co-incubated with extracellular secretions of SAC was unaffected (Figure S8), implying that the biofilm formation was not impeded by altering cell growth and interactions. Here, we observed that the extracellular secretion of SAC inhibited biofilm formation by PAO1 and efficiently disrupted established biofilms through continuous polysaccharide degradation.

### Environmental implications

Membrane fouling remains a major obstacle in the widespread application of MBRs. Biological methods stand out for their sustainable fouling control in practical applications [67]. The composition and structure of a biocake largely depend on its biopolymer content and properties. Thus, the prevention or destruction of biopolymers in biocakes is of great significance for controlling membrane fouling. Previous studies have reported that the cultivation of functional enzymes or bacteria can achieve in situ biodegradation of biopolymers in biocakes [67, 68]. In this study, we use a “top-down” enrichment-based methodology to construct a synthetic anti-fouling consortium. Compared to those of pure cultures, a microbial consortium has a larger gene pool, more diverse metabolic pathways, and utilizes less refined substrates [69]. Syntrophy is a possible interaction that helps metabolize mutually beneficial microbes into communities [14, 70, 71]. Synthetic microbial consortia with controllable functions are expected to exhibit a high degradation efficiency. The purposeful enrichment of a consortium can amplify the dominance of a community, which can be better applied to MBR plants based on their community coalescence with activated sludge. We found that the polysaccharides in the mixed liquor could be effectively degraded by SAC, leading to better filterability of the mixed liquor. However, it is essential to control the appropriate microbial concentration when adding microbial consortium to prevent the occurrence of severe membrane fouling. The indigenous microorganisms have already adapted to the environmental conditions and developed substrate utilization strategies, offering them an ecological advantage [72]. When synthetic microbial communities are introduced into activated sludge, they will compete with the existing indigenous microorganisms for resources, space, and interactions The synthetic microbes must adapt to the new environmental conditions and compete for limited resources such as nutrients and living space. When a small amount of microbial consortium (e.g., 0.1%) was introduced into an existing microbial community, it will be outcompeted by the resident microbes. Due to the limited quantity, the newly introduced microbes may struggle to establish a stable ecological balance with the existing population. The resident microbes are better adapt to the environment and can outcompete the newcomers for limited resources, resulting in the inability of the new population to survive and reproduce.

Besides, two MBRs were operated in parallel under the same constant flux to verify the long-term effect of SAC on fouling evolution. The prolonged operation time in E-MBR demonstrated the considerable biofouling mitigation of the SAC (Text Sb1, Figure S9). The addition of SAC had no adverse effects on nutrients removal in MBRs during the long-term operation (Text Sb2, Figure S9). Therefore, the method proposed in this study provides an alternative to construct a consortium for the achievement of in situ removal of foulants. Nevertheless, the feasibility and activity of SAC should be further evaluated in large-scale investigations or long-term MBR operation.

## Conclusion

The influence of SAC on membrane fouling and the underlying mechanisms were investigated in this study. To sum up, the main conclusions can be drawn as follows:

1. The “top-down” approach resulted in a stable consortium that can effectively degrade alginates. The SAC was dominated by *Massilia*, which could completely degrade alginates after 74 h of cultivation.
2. The anti-fouling mechanism of SAC are attributed to: (a) decreasing the concentrations of polysaccharide in sludge supernatant, (b) damaging the carbohydrate *O*-acetylation in EPS, (c) changing the protein secondary structure associated with bacterial aggregation.
3. The extracellular secretions of SAC can both inhibit biofilm development and disrupt the existing biofilms.

### Electronic supplementary material

Below is the link to the electronic supplementary material.

Supplementary Material 1
