# Supplementary material for: Construction of synthetic anti-fouling consortia: fouling control effects and polysaccharide degradation mechanisms
Source: Microb Cell Fact. 2023 Nov 8;22:230. doi: 10.1186/s12934-023-02235-7 (PMC10631183; doi:10.1186/s12934-023-02235-7)
Supplement: Supplementary file 1 — Supplementary Material 1 [file 12934_2023_2235_MOESM1_ESM.docx]

***Supplementary Information for***

**Construction of synthetic anti-fouling consortia: Fouling control effects and polysaccharide degradation mechanisms**

Ji Qi^a,b^, Qicheng Zhou^a,b^, Danlei Huang ^a,b^, Zhong Yu ^a,b^, Fangang Meng^a,b*^

^a^ School of Environmental Science and Engineering, Sun Yat-sen University, Guangzhou 510275, PR China

^b^ Guangdong Provincial Key Laboratory of Environmental Pollution Control and Remediation Technology (Sun Yat-sen University), Guangzhou 510275, PR China

This supporting information contains 17-page document, including 6-page material

and method descriptions, 6 tables, 9 figures, 5 references and this cover page.

***Material and Methods***

**Text1 DNA Extraction and PCR Amplification.** The samples of synthetic anti-fouling microbial consortium (SAC) were collected by high-speed centrifugation (12,000 rpm, 4°C for 15 min) and then stored at -80°C until DNA extraction and PCR Amplification. Total genomic DNA were extracted from the samples using the DNeasy PowerSoil Kit (Qiagen, Inc., The Netherlands), following the manufacturer’s instructions. DNA were quantified using a NanoDrop spectrophotometer (Thermo Fisher Scientific, Waltham, MA, USA), and the quality of DNA were evaluated by agarose gel electrophoresis. PCR amplification of the bacterial 16S rRNA gene V3-V4 region was performed using primers 338F (5′-ACTCCTACGGGAGGCAGCA-3′) and 806R (5′- GGACTACHVGGGTWTCTAAT-3′). The PCR reaction was conducted according to the following program: initial denaturation at 98°C for 2 min, 25 cycles of 98°C for 15 s, annealing at 55°C for 30 s and extension at 72°C for 30 s, and a final extension of 5 min at 72°C. The PCR products were purified with Agencourt AMPure Beads (Beckman Coulter, Indianapolis, IN), quantified using the PicoGreen dsDNA Assay Kit (Invitrogen, Carlsbad, CA, USA).

**Text2 Assessment of the Unified Modified Fouling Index (UMFI).** The membrane filtration performance and fouling propensity of the SAC-dosed sludge was quantified by UMFI established by Huang et al [1]. UMFI is generally expressed by the following equations:

$$\begin{matrix} J'=\frac{J_{0}}{J} \\ \frac{1}{J'}=1+(UMFI)\times V \end{matrix}$$

Where *J’* is the normalized filtration flux, *J_0_* is the *J* at time zero (L·m^-2^·h^-1^), *V* is the specific permeate volume (L·m^-2^).

**Text3 Preparation for Active Species**

The synthetic anti-fouling microbial consortium (SAC) was precultured in mineral salts medium (MSM) until they reached the exponential growth phase (~ 36 h) and harvested by centrifugation at 6,000 rpm for 10 min at 4°C. The active species of SAC was divided into three fractions: extracellular, periplasmic, and intracellular fractions, and the specific extracted method was showed as below: the cell pellet was then washed twice with freezing ultrapure water, resuspended in 10 mmol·L^-1^ Tris-HCl buffer (10 mL, pH =8.0) and centrifugated (6,000 rpm, 10 min, 4°C). The above supernatants were labeled as the extracellular fraction. Then, cell pellet was resuspended with 25% (v/v) sucrose solution-1mM EDTA and centrifuged (10,000 rpm, 10 min, 4°C). The supernatant was collected as periplasmic fraction by centrifugation at 13,000 rpm for 10 min at 4°C [2]. Then, cell pellet was lysed by a freezing process prior to sonication (225 W, 30min, 4°C, Ultrasonic processor SCIENTZ-950E). Last, cell debris was disposed by centrifugation at 15,000 rpm for 20 min at 4°C, and supernatant was collected as intracellular fraction [3]. The extracellular, periplasmic, and intracellular fractions were filtered through 0.22 *μ*m syringe filter before starting the following degradation experiments. Sodium alginate (SA)was added to three fractions to achieve the final concentration of 100 mg/L, and each sample was incubated the rotary shaker at 150 rpm, 30°C for 74 h. The MSM without SAC was utilized for the control sample. Each experiment was performed in triplicate.

**Text4 *P. aeruginosa* (PAO1) growth assay**

To test the effect of SAC dosage on the inhibition and disruption of biofilm SAC-mediated biofilm inhibition and disruption affect the bacterial growth (*P. aeruginosa*,), the *P. aeruginosa* (PAO1) biofilm was inoculated and then conducted inhibition experiment with extracellular secretions of SAC The bacterial were grown in the rotary shaker at 150 rpm, 30°C. The OD_595_ of each sample was measured for a duration of 24 h at 1-hour intervals. Meanwhile, the cultures without extracellular secretion incubated was used as control.

**Text5 A/O-MBR Setups and Operation**

Two lab-scale A/O-MBR systems with an effective working volume of 9.57 L, volumes of 3 L and 6.57 L were allowed for the anoxic and aerobic tanks, respectively.

Activated sludge from a local full-scale wastewater treatment plant (Zhuhai, China) was used for the startup of anoxic/aerobic tanks. The A/O-MBR systems were continuously operated with the hydraulic retention time (HRT) of 12 h, the sludge retention time (SRT) of 30 days, and the sludge recirculation ratio of 200 %. One reactor coupled with 1% SAC metabolites was set as experimental group (E-MBR), and the other one was set as control group (C-MBR). A flat membrane module (0.1 m^2^, pore size 0.1 *μ*m, PVDF, SINAP-10, SINAP membrane; S&T Co., Shanghai, China) was used to produce treated wastewater. An air diffuser was placed below the membrane module to control the dissolved oxygen (DO) at about 2 mg/L. Once TMP reached approximately 25 kPa, the membrane module was removed and cleaned. The details of synthetic wastewater characteristics are given in Table S1.

**Text6 Two-dimensional correlation spectroscopic (2D-COS) analysis.**

In this study, biodegradation time was used as an external perturbation for the functional group changes of the sodium alginate during the degradation. A set of time-dependent Fourier transform infrared (FTIR) spectroscopy collected above were employed for the 2D correlation analysis based on the work of Noda and Ozaki [4]. According to the Hilbert-Noda transform method, the synchronous (Φ (x1, x2)) and asynchronous (Ψ (x1, x2)) spectra could be generated from the corresponding dynamic spectra as follow (Eqs1,2):

|  | (1) |
| --- | --- |
|  | (2) |

Where is the Hilbert transform of the signal $\mathop{y}\limits^{\sim} (x_{2},t)$, and $t^{'}$ means the integral variables. The value of the $\Phi(x_{1},x_{2})$ indicates the simultaneous or coincidental changes of spectral intensity variations observed at $x_{1}$ and $x_{2}$ during the interval between $T_{\min}$ and $T_{\max}$. The $\psi(x_{1},x_{2})$ represents the asynchrony of spectral intensity variations measured at $x_{1}$ and $x_{2}$ which can reveal the sequence of the variable changes caused by the given perturbations. Briefly, (1) if the intensity of synchronous spectrum is positive ($\Phi(x_{1},x_{2})$>0), and the same intensity of the asynchronous spectrum ($\psi(x_{1},x_{2})$>0) indicates that the change at peak$x_{1}$ occurs before those at peak $x_{2}$. In the contrast, the negative cross peak ($\psi(x_{1},x_{2})$ < 0) reveals that the change at $x_{1}$ occurs after $x_{2}$ (2) if the intensity of the synchronous spectrum is negative ($\Phi(x_{1},x_{2})$ < 0), the above rule is inverted. (3) if $\Phi(x_{1},x_{2})$ or $\psi(x_{1},x_{2})$ is zero, the change at $x_{1}$,$x_{2}$ occur concurrently. Prior the 2D CoS analysis, the FTIR spectra was normalized, baseline-corrected and denoised by the ONMIC 7.3 (Thermo Fisher Scientiﬁc Co., USA). The 2D-CoS computation was conducted with the 2Dshige software (Kwansei-Gakuin University, Japan).

**Text7 Contact Angle Measurement**

Homogenous sludge layers were prepared by gathering the sludge cells on the 0.22 *μ*m acetate cellulose membrane, which were washed three times with the PBS solution (pH=7.2) then and placed on the 1 % agar plate [5]. Before the measurement, the membranes with sludge layers were mounted on the coverslip and air-dried for 24 h. After that, 2 *μ*l deionized water was dropped onto the membrane. All measurements were repeated at least 5 times.

***Results and discussion***

**Text b1Application of SAC to the MBR performance**

Considering the potential effects of SAC on microbial population dynamics and gene transcription, the nutrient removal performance in both MBRs were recorded during the operation (Figure S9). With the same influent, excellent nitrification was obtained with an average removal efficiency of TN of 65.05% and 62.8% in the control and experimental group, respectively (Figure S9d). Similarly, COD removal efficiency in the control and experimental group were 84.92% and 86.19%, respectively (Figure 9c). Collectively, it can be concluded that SAC metabolites had no adverse effects on nutrients removal in MBRs during the long-term operation.

**Text b2 SAC metabolites effects on the EPS relevant to biofouling**

To verify the long-term effect of SAC on fouling evolution, two MBRs were operated in parallel under the same constant flux. TMP was monitored to quantitatively evaluate the extent of biofouling in MBRs (Figure S9). At the stable stage, in C-MBR, it took 16.17 days to reach the TMP of 50 kPa, whereas it took 22.40 days in the E-MBR with SAC metabolites to reach the same TMP. The prolonged operation time in E-MBR demonstrated the considerable biofouling mitigation of the SAC.

**Table S1.** The media composition of MSM used in this study.

| **Components** | **Concern (mg/L)** |
| --- | --- |
| KH_2_PO_4_ | 500 |
| K_2_HPO_4_·3H_2_O | 1,910 |
| NH_4_NO_3_ | 1,500 |
| MgSO_4_·7H_2_O | 200 |
| NaCl | 500 |

**Table S2.** Trace elements solution composition of MSM used in this study. [6]

| **Components** | **Concern (mg/L)** |
| --- | --- |
| CoCl_2_·6H_2_O | 100 |
| MnCl_2_·4H_2_O | 425 |
| ZnCl_2_ | 50.0 |
| NiCl_2_·6H_2_O | 1.00 |
| CuSO_4_·5H_2_O | 15.0 |
| Na_2_MO_4_·2H_2_O | 10.0 |
| Na_2_SeO_4_2H_2_O | 10.0 |

**Table S3.** Components and concentration of synthetic wastewater.

|  | **Substrates** | **Concern (mg/L)** | **Trace elements** | **Concern (mg/L)** |
| --- | --- | --- | --- | --- |
| Ingredients | K_2_HPO_4_ | 17.56 | FeSO_4_ 7H_2_O | 2.5 |
|  | NH_4_Cl | 152 | ZnCl_2_ | 0.06 |
|  | Glucose | 280 | MnCl_2_ 4H_2_O | 0.06 |
|  | Peptone | 82 | Na_2_MoO_4_ 2H_2_O | 0.19 |
|  | Beef extract | 28 | CoCl_2_ 6H_2_O | 0.13 |
|  | NaHCO_3_ | 20 | NiCl_2_ 6H_2_O | 0.04 |
|  |  |  | CuSO_4_ | 0.06 |
|  |  |  | H_3_BO_3_ | 0.06 |
|  |  |  | MgCl_2_ 6H_2_O | 0.19 |
|  |  |  | CaCl_2_ | 0.44 |

**Table S4.** Assignment and signs of cross peaks in Synchronous (Φ) and Asynchronous (Ψ, in the Brackets) Maps of SA biodegradation by SAC within 74 h

|  |  | **Sign** | | | | | | | | | | | | | | | | | |
| --- | --- | --- | --- | --- | --- | --- | --- | --- | --- | --- | --- | --- | --- | --- | --- | --- | --- | --- | --- |
| **peak (cm^−1^)** | **assignment** | 1725 | 1650 | 1604 | 1406 | 1317 | 1294 | 1205 | 1161 | 1137 | 1122 | 1103 | 1056 | 1018 | 991 | 956 | 916 | 885 |
| 1725 | COOH, -C=O | + | + (+) | + (+) | + (+) | + (+) | + (+) | +（-） | +（+） | - (+) | - (+) | - (+) | +（-） | + (+) | + (+) | + (+) | + (+) | + (+) |
| 1650 | asymmetric C−O stretching of COOH |  | + | + (+) | + (+) | +（0） | +（0） | +（0） | - (+) | +（-） | +（-） | - (-) | - (+) | + (+) | +（-） | +（0） | +（0） | + (+) |
| 1604 | asymmetric vibration of COOH |  |  | + | + (+) | +（0） | +（-） | + (+) | -（-） | -（-） | -（-） | -（-） | +（0） | + (+) | +（0） | + (+) | +（0） | +（0） |
| 1406 | symmetric vibration of COOH |  |  |  | + | +（-） | +（-） | + (+) | -（-） | -（-） | -（-） | -（-） | +（-） | + (+) | +（-） | + (+) | +（0） | +（-） |
| 1317 | asymmetric C−O stretching of COOH |  |  |  |  | + | +（-） | +（-） | +（0） | -（+） | -（+） | -（+） | -（+） | + (+) | +（-） | +（-） | +（-） | +（-） |
| 1294 | vibration of C−O−C bonds |  |  |  |  |  | + | +（-） | +（+） | +（0） | +（0） | -（-） | -（0） | + (+) | + (+) | +（0） | +（0） | +（0） |
| 1205 | vibration of C−O−C bonds |  |  |  |  |  |  | + | + (+) | + (+) | -（+） | -（+） | -（-） | +（-） | +（-） | +（-） | +（-） | +（-） |
| 1161 | vibration of C−O−C bonds |  |  |  |  |  |  |  | + | +（+） | +（-） | +（-） | - (+) | - (+) | - (+) | -(+) | -(+) | +(-) |
| 1137 | vibration of C−O−C bonds |  |  |  |  |  |  |  |  | + | +（0） | +（+） | + (+) | - (-) | -(+) | -(0) | -(0) | -(-) |
| 1122 | vibration of C−O−C bonds |  |  |  |  |  |  |  |  |  | + | +（-） | + (+) | - (+) | -(+) | -(-) | -(0) | -(-) |
| 1103 | vibration of C−O−C bonds |  |  |  |  |  |  |  |  |  |  | + | - (+) | -(0) | - (+) | -(0) | -(+) | -(-) |
| 1056 | antisymmetric C−O−C stretching |  |  |  |  |  |  |  |  |  |  |  | + | + (+) | + (-) | +（+） | +（-） | +（-） |
| 1018 | C-O-C |  |  |  |  |  |  |  |  |  |  |  |  | + | +（-） | +（0） | +（-） | +（-） |
| 991 | C-O stretching of uronic acids |  |  |  |  |  |  |  |  |  |  |  |  |  | + | + (+) | +（0） | +（0） |
| 956 | C-O stretching of uronic acids |  |  |  |  |  |  |  |  |  |  |  |  |  |  | + | +（0） | +（0） |
| 916 | C-O stretching of uronic acids |  |  |  |  |  |  |  |  |  |  |  |  |  |  |  | + | + (-) |
| 885 | C−H deformation |  |  |  |  |  |  |  |  |  |  |  |  |  |  |  |  | + |

*Note: (i) “+” means positive sign; “-” means a negative sign; “0” denotes no peak. (ii) * denotes that signs were obtained in the upper-left corner of the 2D maps.

**Table S5**. Changes in water contact angle (WCA) and zeta potential after the microbial consortia were incubated in the sludge

| Surface characteristic | | |
| --- | --- | --- |
| group | Zeta potential (mV) | Water contact angle (°) |
| 0% | -20.50 ± 1.06 | 78.65 ± 5.73 |
| 0.1% | -16.78 ± 0.60 | 84.48 ± 5.08 |
| 0.5% | -14.87 ± 0.23 | 86.73 ± 1.16 |
| 1% | -14.18 ± 1.19 | 89.93 ±1.98 |
| 2% | -11.91± 0.76 | 88 ± 2.38 |
| 5% | -10.9 ± 0.64 | 87.74 ± 3.16 |
| 10% | -10.89 ± 0.29 | 88.74 ± 1.36 |

**Table S6.** Band assignments and functional groups for the FTIR spectral features (cm-^1^) of EPS from different groups

| **Group** | **O-H stretching (hydrogen-bonded)** | **C-H stretching (CH_2_ and CH_3_)** | **COOH, C=C** | **C=O stretch**  **(amide I)** | **C-H, C-N**  **(amide Ⅱ)** | **CH_2_** | **C=O**  **(amide II)** | **C-N**  **(amide III)** | **uronic acids** | **C-O-C stretching of uronic acids** | **C-H deformation** |
| --- | --- | --- | --- | --- | --- | --- | --- | --- | --- | --- | --- |
|  | **~ 3300** | **~ 2900** | **~ 1700** | **~ 1600** | **~ 1500** | **~ 1440** | **~ 1400** | **1300-1200** | **1040-1010** | **910-970** | **~ 880** |
| 0% |  |  |  |  |  |  |  |  |  |  |  |
| SAC |  |  |  |  |  |  |  |  |  |  |  |
| 0.1 % |  |  |  |  |  |  |  |  |  |  |  |
| 0.5 % |  |  |  |  |  |  |  |  |  |  |  |
| 1 % |  |  |  |  |  |  |  |  |  |  |  |
| 2 % |  |  |  |  |  |  |  |  |  |  |  |
| 5 % |  |  |  |  |  |  |  |  |  |  |  |
| 10 % |  |  |  |  |  |  |  |  |  |  |  |

Note: Box with pink color represents the presence of relevant functional group in EPS


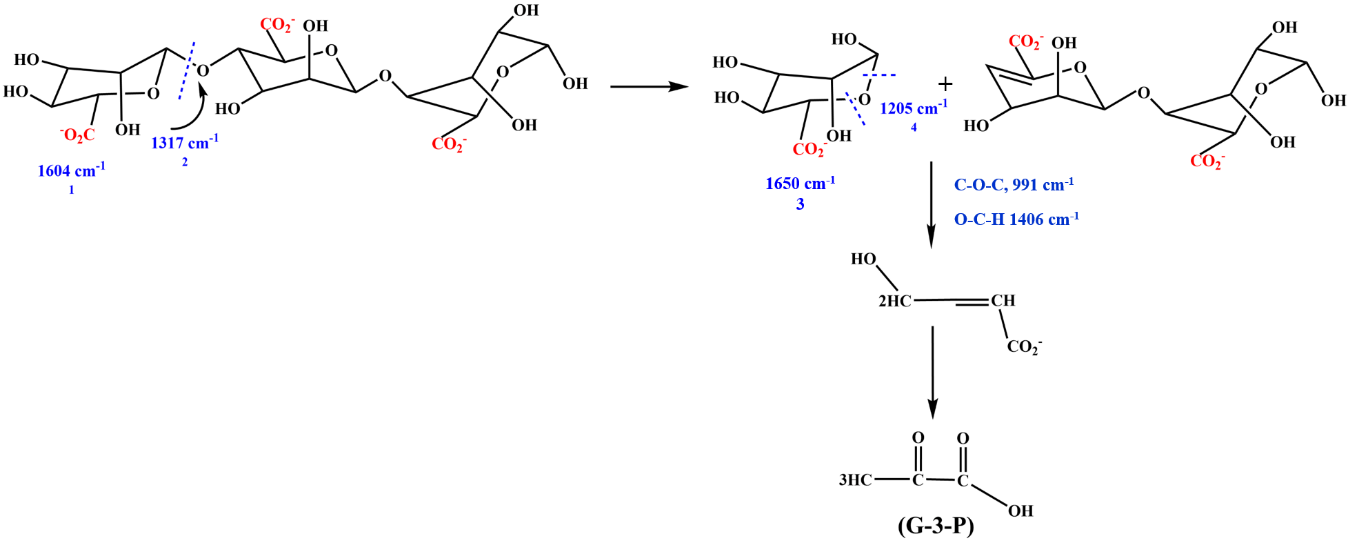


**Figure S1**. **the biodegradation process of polysaccharide by SAC**


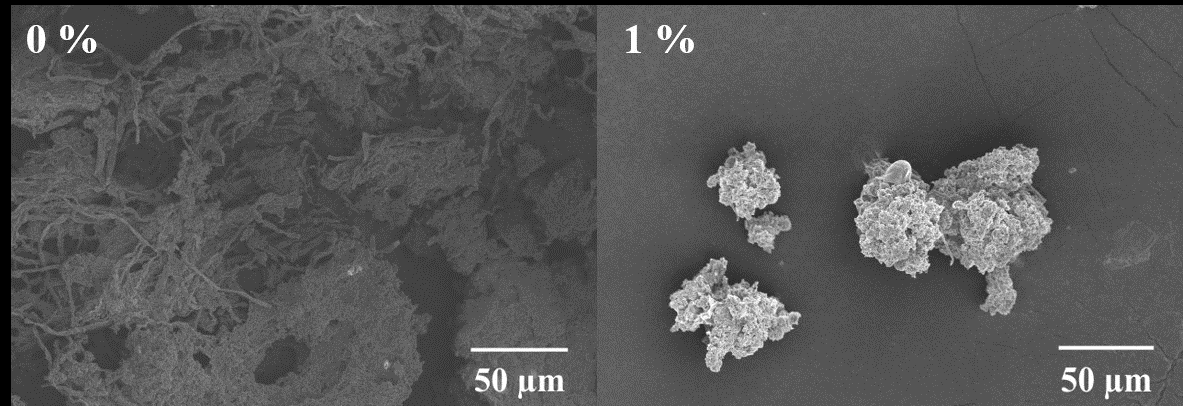


**Figure S2**. **Morphological characterizations of membranes after the dead-end filtration.** SEM surface micrographs of 0% (left) and 1% SAC-dosed sludge(right). Scale bars in SEM are 50 *μ*m.


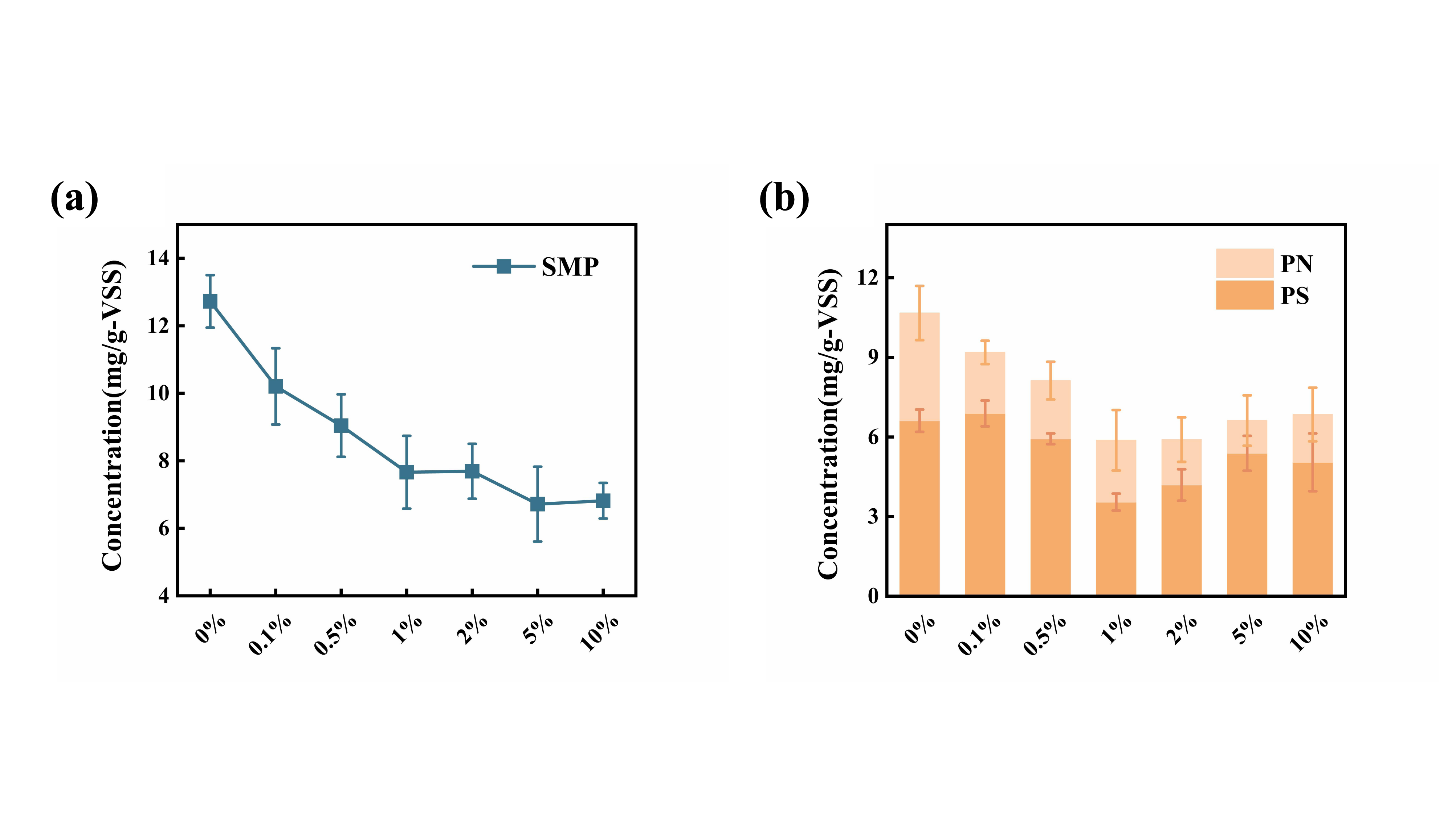


**Figure S3. The characterization of TOC, proteins (PN) and polysaccharides (PS) in the SAC-dosed sludge (a)** TOC concentration in SMP of activate sludge with different dosed concentrations of microbial consortia, **(b)** The proteins (light orange columns) and polysaccharides (deep orange columns) contents of SMP from the sludge. Error bars represent the standard deviations of triplicate.


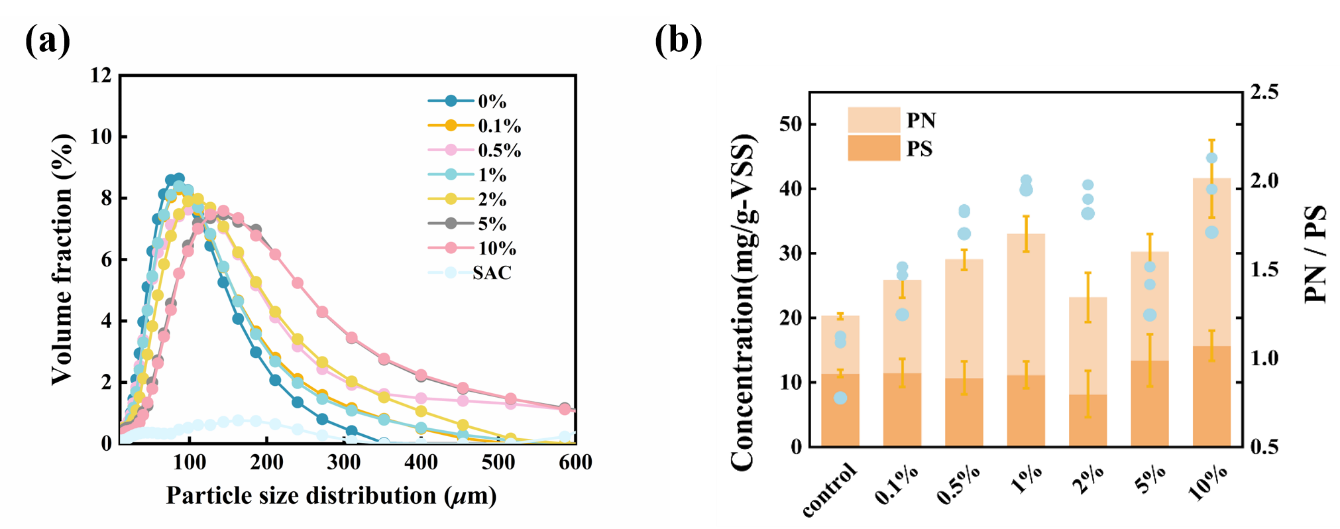
**Figure S4. The characterization of particle distribution, proteins (PN) and polysaccharides (PS) in the SAC-dosed sludge (a)** Particle size distribution of bulk sludge collected from different SAC-dosed group, **(b)** Concentrations of PN and PS of EPS from the sludge


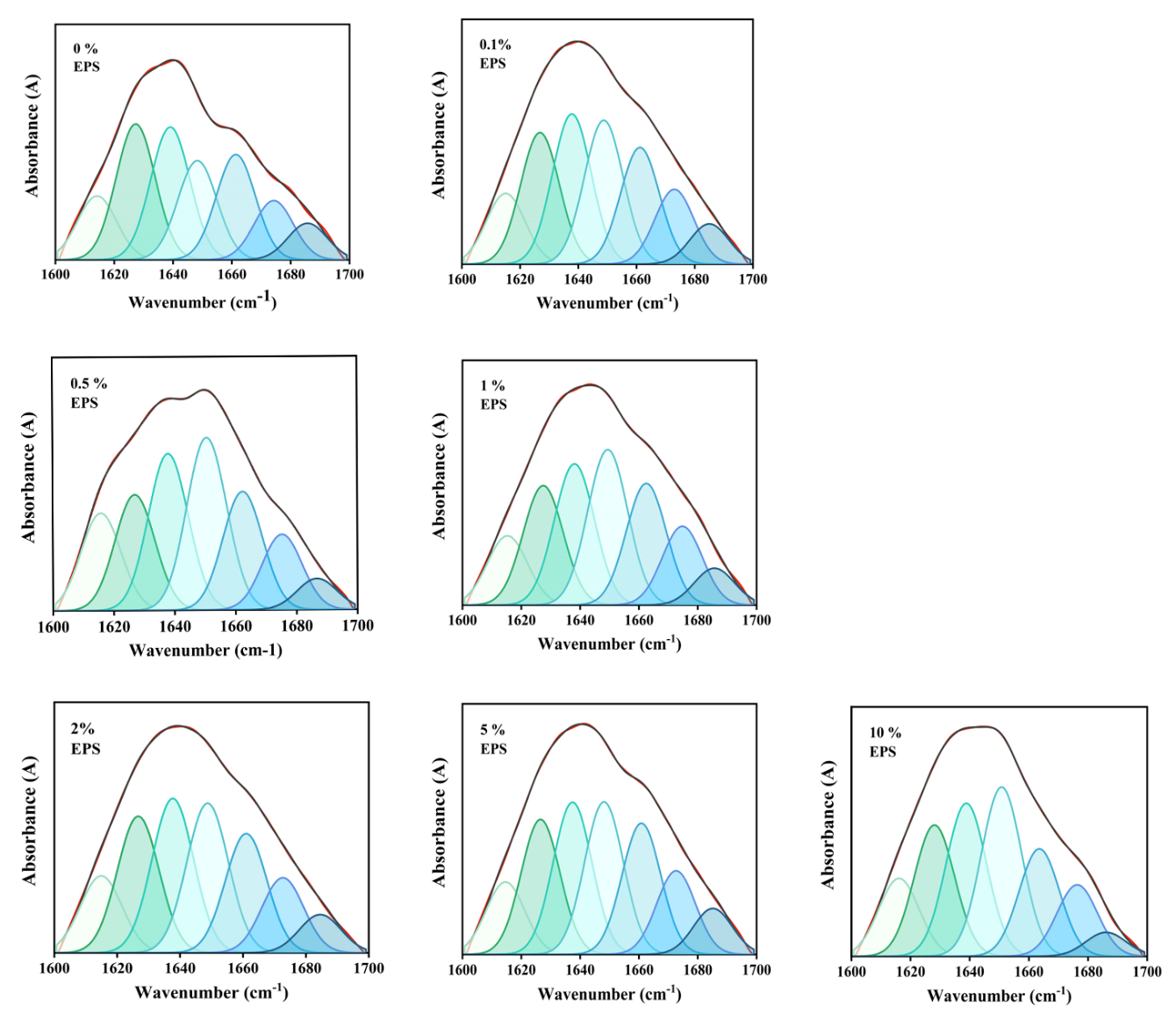


**Figure S5.** Second derivative resolution enhancement and curve-fitted amide I region (1700-1600 cm^-1^) of protein from EPS extracted from different SAC-incubated groups.


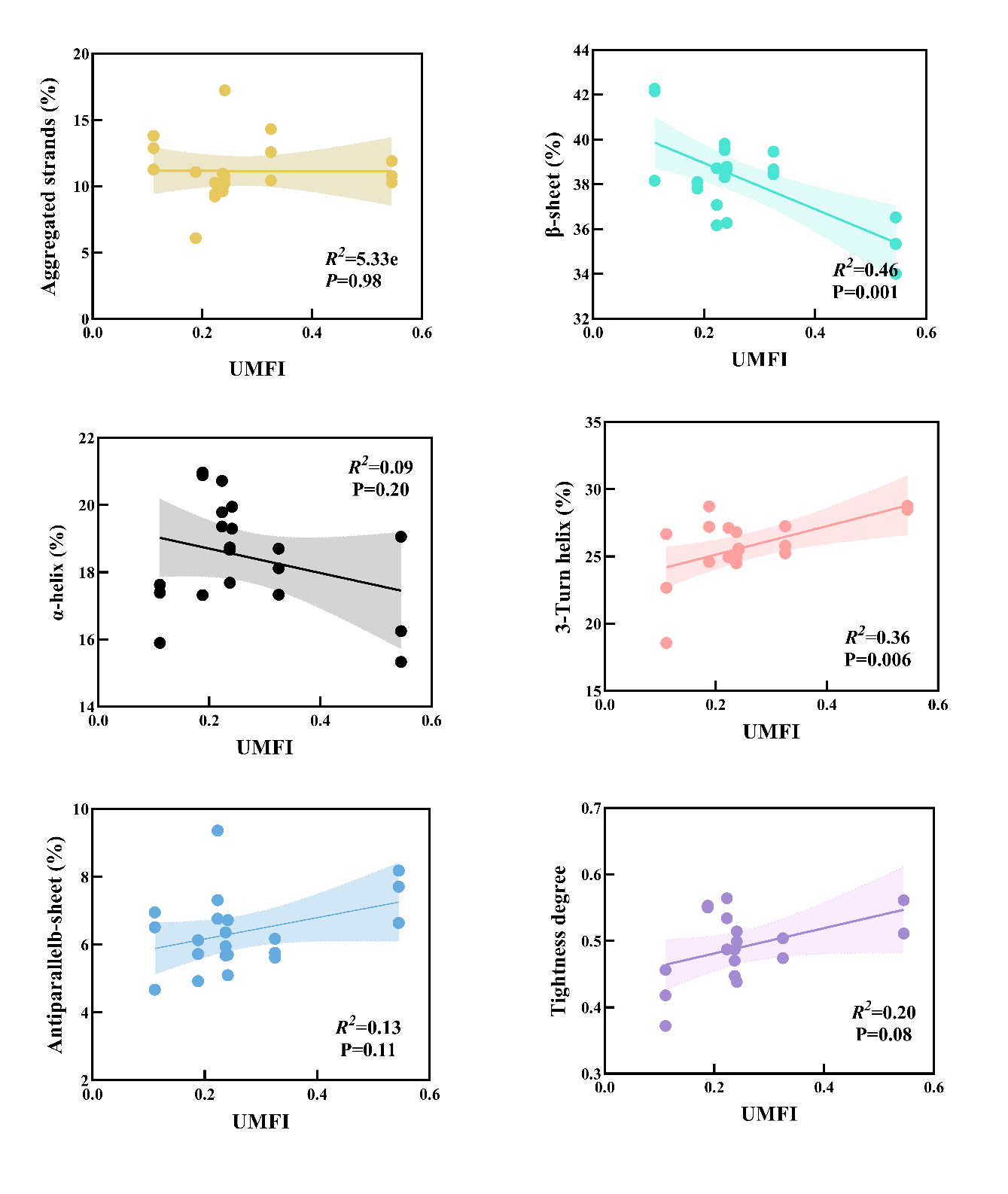


**Figure S6.** Correlation analysis (fit line with liner regression) between the protein secondary structure in the SAC dosed EPS and membrane fouling potential index (UMFI)


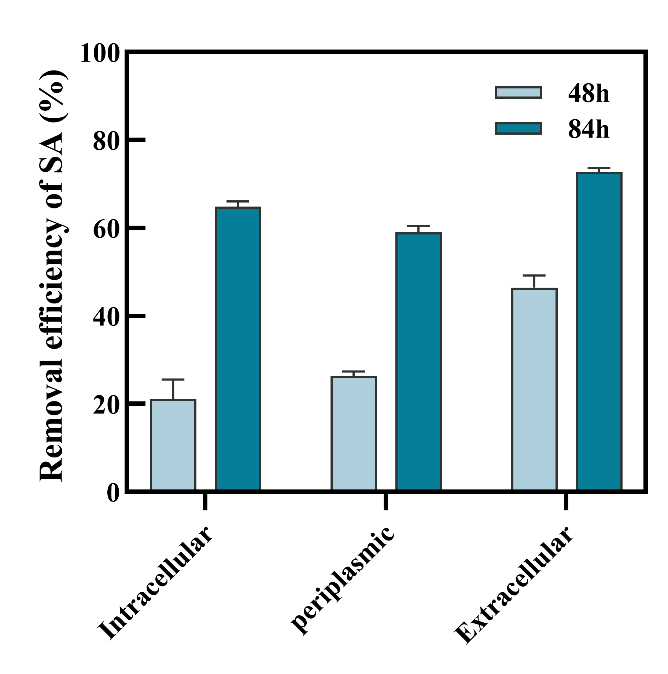


**Figure S7.** Degradation efficiency of SA (100 mg⋅L^−1^) by the extracellular, periplasmic, and intracellular fractions of SAC in the MSM after 84 h of cultivation. Error bars represent the standard deviations of triplicate.


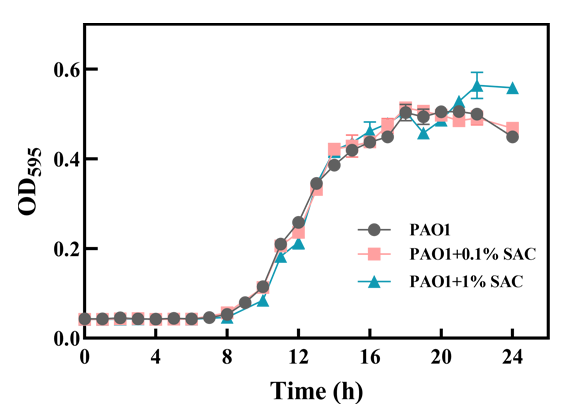


**Figure S8.** The growth curve of PAO1 in LB broth with various concentration of the extracellular fraction of SAC (24 h, 30 ℃). Error bars represent the standard deviations of triplicate.


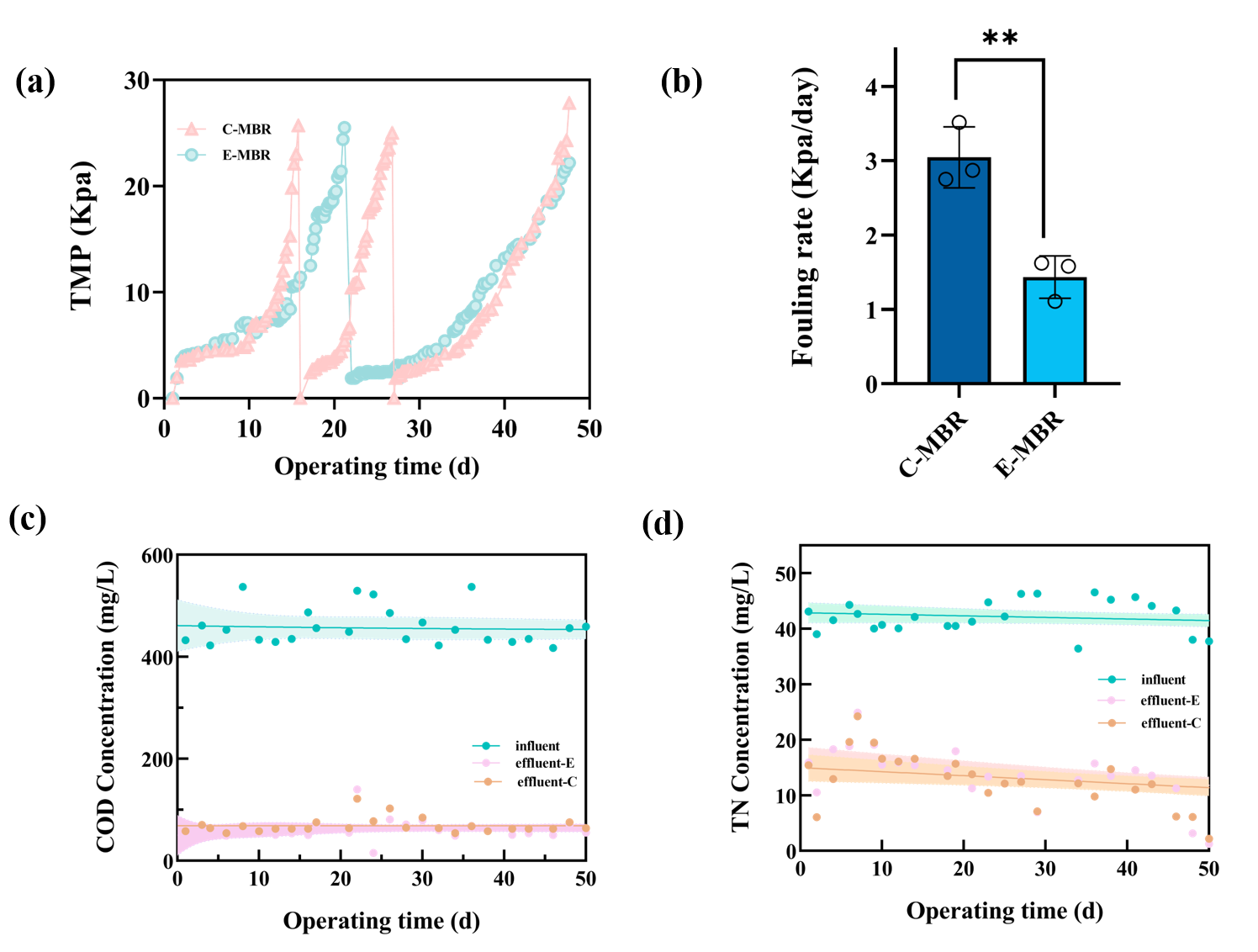


**Figure S9**. **Mitigation of biofouling by SAC and the reactor operation.** Dynamic profiles of TMP (a) and its increasing rate (b) in the C-MBR and E-MBR over the long-term operation (c) COD and (d) TN removal during the reactor operation**.**

**References**

1. Huang H, Young TA, Jacangelo JG. Unified Membrane Fouling Index for Low Pressure Membrane Filtration of Natural Waters: Principles and Methodology. Environ Sci Technol. 2008;42:714–20.

2. Park SJ, Lee SY. Efficient recovery of secretory recombinant proteins from protease negative mutant Escherichia coli strains.

3. Gu C, Wang J, Guo M, Sui M, Lu H, Liu G. Extracellular degradation of tetrabromobisphenol A via biogenic reactive oxygen species by a marine Pseudoalteromonas sp. Water Res. 2018;142:354–62.

4. Noda I. Chapter 13 - Generalized Two-Dimensional Correlation Spectroscopy. In: Laane J, editor. Front Mol Spectrosc [Internet]. Amsterdam: Elsevier; 2009 [cited 2023 Apr 7]. p. 367–81. Available from: https://www.sciencedirect.com/science/article/pii/B9780444531759000131

5. Liu X.-M, Sheng G.-P, Luo H.-W, Zhang F, Yuan S.-J, Xu J, Zeng R.J, Wu J.-G, Yu H.-Q. Contribution of Extracellular Polymeric Substances (EPS) to the Sludge Aggregation. Environ. Sci. Technol. 2010;44: 4355–4360.

6. Cove DJ. The induction and repression of nitrate reductase in the fungus Aspergillus nidulans. Biochim Biophys Acta BBA - Enzymol Biol Oxid. 1966;113:51–6.
